# Marine-derived carbon dots: a safe and effective solution for noise-induced hearing loss

**DOI:** 10.1186/s12951-026-04282-9

**Published:** 2026-03-16

**Authors:** Guangsen Xu, Jing Wang, Guige Hou, Xiaoya Wang, Yuliang Xu, Jiajun Tian, Yanjiao Ding

**Affiliations:** 1 School of Pharmacy, the Key Laboratory of Prescription Effect and Clinical Evaluation of State Administration of Traditional Chinese Medicine of China, Binzhou Medical University, Yantai, 264003 People’s Republic of China; 2 Department of Pharmacy, The Second Qilu Hospital of Shandong University, 247 Beiyuan Street, 250033 Jinan, Shandong People’s Republic of China; 3 Department of Otolaryngology-Head and Neck Surgery, Shandong Provincial ENT Hospital, Shandong University, Jinan, Shandong People’s Republic of China; 4 Department of Pharmacy, Shandong Second Provincial General Hospital, Jinan, Shandong People’s Republic of China

**Keywords:** Carbon dots, Marine resources, Noise-induced hearing loss, Blood-labyrinth barrier crossing, Reactive oxygen species, Ferroptosis

## Abstract

Noise-induced hearing loss (NIHL) remains highly prevalent in occupational settings, and no pharmacological agent or combination thereof has demonstrated a protective or restorative impact against noise-induced damage in clinic till now. Biomass-derived carbon dots (CDs) are known for their unique biological activities and excellent safety. Marine organisms account for 78% of the planet’s biomass. In this vast and diversified habitat, marine animals make and gather significant amounts of compounds with distinct structural and physiological properties. Here, using eight different marine fauna and flora as carbon sources, safe and green CDs were synthesized through one-pot hydrothermal method. All eight CDs displayed good stability and radical quenching abilities. In ABR testing, the *Undaria pinnatifida* Suringar-derived CDs (USCDs) exerted the strongest hearing protection abilities under noise exposure *in vivo* among the eight samples. The structure–activity relationships of marine-derived CDs suggested that improved content of heteroatom or reductive function groups was essential for their radical scavenging and *in vivo* anti-NIHL activities. The cytoprotective activity for auditory nerve and hair cells *in vivo* were confirmed by confocal microscopy. Notably, *in vitro* cell tests demonstrated that USCDs could prevent apoptosis and ferroptosis through ROS scavenging and Fe^2+^-chelating, respectively. Moreover, both *in vivo* and *in vitro* experiments demonstrated that USCDs possessed excellent biosafety and blood–labyrinth barrier (BLB) permeability, supporting their potential for systemic administration. These findings broaden the potential applications of CDs in the treatment of inner ear diseases, and provided some insights into the development marine drugs.

**Graphical Abstract:**

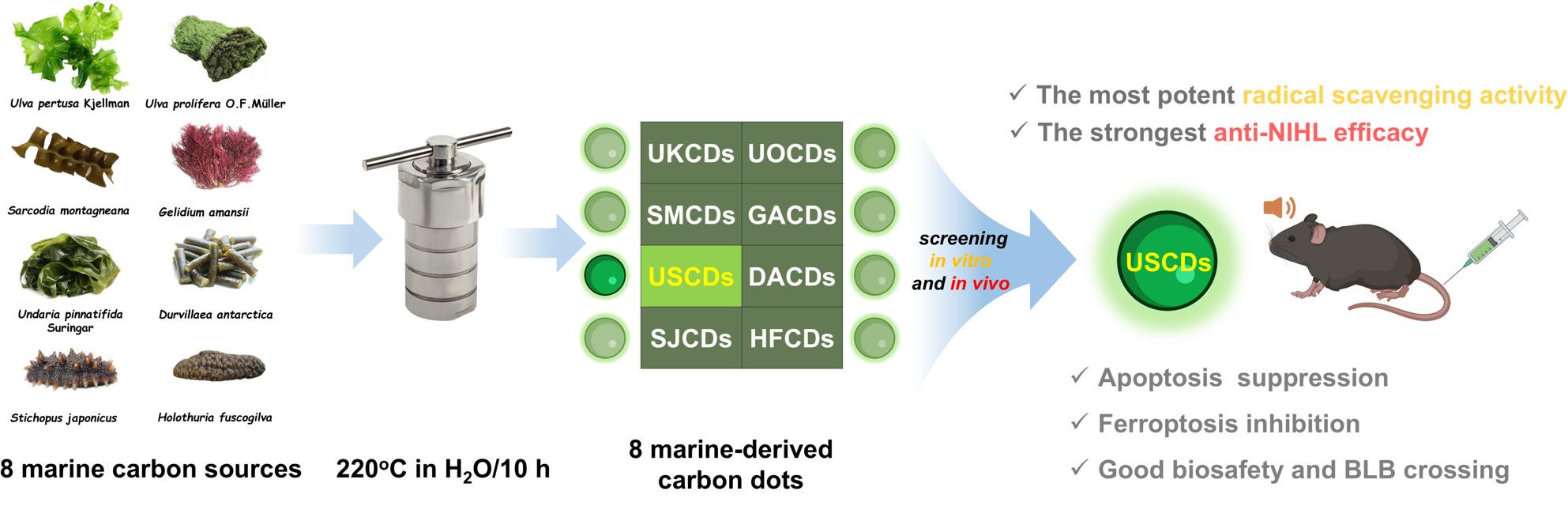

**Supplementary Information:**

The online version contains supplementary material available at 10.1186/s12951-026-04282-9.

## Introduction

The World Health Organisation reports that 466 million individuals globally experience sensorineural hearing loss, representing over 50% of the population aged 60 and above [1, 2]. In both developing and industrialised nations, excessive noise (> 70 dB SPL) accounts for 50% of auditory system damage and is the predominant preventable cause of hearing loss (HL), second only to presbycusis. Mitigating noise-induced hearing loss (NIHL) presents sociocultural challenges, including inadequate noise regulation, social tolerance of elevated noise levels, a lack of awareness of its impact on quality of life, excessive dependence on hearing protection, and challenges in identifying early signs of hearing impairment. Multiple techniques are being researched for the prevention and healing of harm caused by exposure to excessive noise from industrial or recreational activities [3]. However, despite recent progress in understanding the genetic and molecular mechanisms underlying NIHL, no pharmacological agent or combination has yet demonstrated clinically effective protective or restorative benefits against noise-induced auditory injury [4].

Permanent NIHL occurs after high-intensity exposure (> 100 dB SPL) or repeated overstimulation. In this scenario, irreversible HL may occur due to cochlear hair cell (HC) loss or permanent injury to mechanosensory structures. Oxidative stress is strongly linked to the progression of various diseases, including age-related macular degeneration, Parkinson’s disease, alcoholic hepatitis, and cardiovascular diseases [5–7]. Although multiple hypotheses have been proposed to explain NIHL pathogenesis, accumulating evidence indicates that the key mechanisms include cochlear inflammation, oxidative stress induced by reduced cochlear blood flow and excessive reactive oxygen species (ROS) production, as well as elevated intracellular calcium levels [8, 9]. The disrupted signaling cascade disrupts mitochondrial function and activates apoptotic pathways, DNA breakage, and cell death [4, 10]. Based on said evidence, antioxidants, anti-apoptotic and anti-inflammatory agents have become the most studied promising tools against NIHL [10]. More recently, ferroptosis—an iron-dependent form of regulated cell death driven by lipid peroxidation—has been identified as another critical mechanism underlying hair cell loss, thereby providing a new theoretical basis for the prevention and treatment of NIHL [11–14].

Hearing in humans relies on mechanosensitive hair cells located in the organ of Corti. Mammalian hair cells are post-mitotic, terminally differentiated sensory cells of the inner ear, and the loss of hair cells is irreversible [15, 16]. Thus, early treatment and preventing are critical to NIHL. Compared with short cycle therapy, the medication for NIHL prevention needs higher security reliability. Furthermore, due to severe restriction of the blood-labyrinth barrier (BLB), lack of sufficient drug concentrations in the inner ear often exists after intravenous injection or oral intake (systemic administration), which makes inner ear therapy particularly challenging [17, 18]. Hence, development of novel safe and effective preventive agents exerting BLB crossing is of significance for coping with NIHL.

Carbon dots (CDs), a novel category of carbon-based nanoparticles, demonstrate exceptional fluorescent characteristics, elevated water solubility, high metabolic stability and commendable biocompatibility. The characteristic features of CDs have been the potential tools for *in vivo* imaging and disease treatments [19, 20]. In particular, biomass-derived CDs synthesized via hydrothermal processes have attracted considerable attention, as their surfaces often retain functional groups from the precursor materials, thereby preserving fraction of the intrinsic bioactivity of the original biomass. Compared with CDs prepared from chemical reagents or organic solvents, biomass-derived CDs also minimize the risks associated with residual solvent or reagent toxicity. Owing to these advantages, biomass-derived CDs are increasingly regarded as promising theranostic nanomedicines, with demonstrated potential in diverse biomedical applications, including antibacterial, antiviral, and anti-gout therapies [21–24].

Marine organisms represent approximately 78% of the Earth’s total biomass [25]. Within this vast and diverse ecosystem, marine species synthesize and accumulate a wide range of bioactive compounds—such as fucoidan [26], agarose [27], and sea cucumber saponin [28]—characterized by unique structural features and diverse physiological activities, which provide a valuable foundation for modern drug discovery and development [29]. The long-standing tradition of seafood consumption among coastal populations further supports the safety of marine-derived resources. Accordingly, exploiting marine biomass as precursors for the synthesis of bioactive carbon dots (CDs) not only holds considerable promise for pharmaceutical innovation but also contributes to the sustainable growth of the marine economy [30].

In this work, eight marine-derived CDs were synthesized through the one-pot hydrothermal method [31] on the basis of six macroalgae (green algae: *Ulva pertusa* Kjellman and *Ulva prolifera* O. F. Müller; red algae: *Sarcodia montagneana* and *Gelidium amansii*; brown algae: *Undaria pinnatifida* Suringar and *Durvillaea antarctica*) and two echinoids (*Stichopus japonicus* and *Holothuria fuscogilva*). The eight precursors encompass diverse natural polysaccharides with structural diversity, antioxidant [32–34] and neurotrophic activities [35, 36]. Among the 8 CDs, the USCDs derived from *Undaria pinnatifida* Suringar exhibited the highest free radical scavenging efficacy and strongest anti-NIHL activity *in vivo* with excellent biosafety and biocompatibility.

## Materials and methods

### Preparation of marine-derived CDs

4 g of marine macroalgae or echinoids was placed in 40 mL of deionised water as a prelude. The mixture was placed in a stain less steel autoclave with a Teflon-lined interior and heated to 220 °C with magnetic stirring for 10 h to achieve a homogenous solution. Upon naturally cooling to room temperature (25 °C), the resultant solutions were subjected to filtration through a 0.2 μm membrane filter to eliminate larger aggregates. The products underwent purification utilising a 500 Da dialysis bag for 72 h to eliminate unreacted small compounds. Following filtration with a membrane filter, the marine-derived carbon quantum dots were acquired.

### Characterization methods

Transmission electron microscopy (TEM) imaging of marine-derived CDs was performed on a FEI Tecnai F20 microscope. The Fourier transform infrared (FTIR) spectroscopy was carried out using a Nicolet iS 10 FTIR spectrometer. The photoluminescence (PL) spectroscopy was performed by a F-2700 (HITACHI, Japan) luminescence spectrometer. The X-ray photoelectron spectroscopy (XPS) was performed using a Thermo K-alpha spectrometer with Mg Kα X-ray (*hv* = 1283.3 eV).

### EPR test to assess ROS quenching activity of CDs

The purchased graphite phase carbon-nitride-g-C3N4 was dispersed in methanol and configured at a concentration of 0.5 mg/mL. Pipette 50 µL of the solution, add 10 µL of pure DMPO and 40 µL of methanol, mix well, and irradiate with a 300 W xenon lamp for 5 min. Collect the data as control data. The experimental sample group only needed to replace 40 µL of methanol with 40 µL of sample dispersion at varying concentrations. The hydroxyl radicals and singlet oxygen scavenging activities created in the same way as previously explained. Detection of hydroxyl radicals and singlet oxygen by EPR Spectroscopy DMPO (0.1 g/mL in ultrapure water) and TEMP (0.1 g/mL in ultrapure water) were used to capture the hydroxyl radicals and singlet oxygen, respectively.

### Animals

Wild type C57BL/6 mice were 8–10 weeks old and weighed about 16–24 g, and they were purchased from the Animal Center of Shandong University in the city of Jinan in China. They were housed at an appropriate temperature of about 20–22℃ and humidity of about 55–65% with light/dark cycles for 12/12 h and free access to water and feed. The whole animal handling procedures were performed in accordance with the National Institutes of Health Guide for the Care and Use of Laboratory Animals and approved by the Animal Care Committee of Shandong University (No. ECAESDUSM 20,123,011). Throughout the whole experiments, anesthesia was induced using a 3% isoflurane–oxygen mixture in an induction chamber, and subsequently maintained with 0.5%–2% isoflurane in oxygen at a flow rate of 1 L/min throughout the experimental procedures. The mice were anesthetized and maintained at a constant core body temperature throughout the procedure using a warming blanket. All efforts were made to minimize the number of animals used and to alleviate any potential discomfort in the study.

### Noise exposure

Mice were awake and unrestrained, and placed in a separate wire mesh cage on a platform in a sound-proof chamber in this study. The sound-proof room chamber was fitted with a DR 45 N driver (Visaton, Germany) operated by a function/arbitrary waveform generator (Agilent 33210 A) and the output of the sound card was amplified (Crown CDi1000, AVIS). The driver was positioned 5–10 cm above the animals’ vertex, which were exposed to an octave-band noise (4–32 kHz) at 105 dB sound pressure level (SPL) for 2 h. Noise calibration was performed prior to every single exposure session.

### Marine-derived CDs injection

To evaluate the roles for CDs in noise-induced hearing loss, mice were pretreated through intravenous injection with eight CDs (10 mg/kg) or equivalent NS two days before noise challenge. In all cases, hearing was tested on 2 days after noise exposure. After then, those mice were anesthetized by 0.5%–2% isoflurane in oxygen at a flow rate of 1 L/min and cochlear were extracted for immunofluorescence analysis. To observe the long-term efficacy of USCDs, the hearing levels were continuously monitored up to 7 days and 14 days.

### Auditory function evaluation

Auditory brainstem response (ABR) tests were performed for assessing auditory function in mice. Mice were housed in soundproofing chambers and anesthetized with an 0.5%–2% isoflurane‑oxygen mixture, meanwhile they were maintained at a constant core body temperature throughout the procedure using a warming blanket. ABR waveforms were recorded by using subdermal needles placed in the skull, below the pinna, and on the back of mice in a sound isolation chamber and measured with the TDT System 3 (Tucker-Davis Technologies, Alachua, FL, USA). Mice were stimulated at the sound frequencies (4, 8, 12, 16, 24, and 32 kHz) and repeated 1,024 times at each frequency. The sound level began from 90 dB and then reduced by 5 dB sound pressure level (SPL) to the acoustic threshold. ABR threshold was determined by the presence of at least 3 of the 5 waveform peaks about the minimal SPL resulting in a reliable ABR recording. The auditory brainstem response (ABR) thresholds were recorded at the specified stimulation frequency, and visual inspection was conducted to identify one or more distinguishable waves. This process was repeated to ensure the consistency of the waveforms.

### Tissue preparation and immunofluorescence analyses

For immunofluorescence analysis of cochlear hair cells and synapses, dissected mice cochleae were fixed in 4% paraformaldehyde (PFA) for 24 h at 4 °C, permeabilized with 1% Triton X-100 in phosphate-buffered saline (PBS, pH 7.4) for 30 min at RT, and blocked with 5% normal donkey serum in PBS containing 0.1% Tween-20 for 1 h at room temperature (RT). Subsequently, tissues were incubated overnight at 4 °C with primary antibodies targeting myosin 7a (1:400; Cat# 25–6790, Proteus Biosciences) or CtBP2 (1:200; Cat# 612,044, BD Biosciences) diluted in blocking solution. Following three 10-min PBS washes, samples were incubated for 2 h at RT with species-specific Alexa Fluor-conjugated secondary antibodies (1:1,000; Invitrogen) and DAPI nuclear stain (1:1,000; Cat# D9542, Sigma-Aldrich) in darkness. After extensive washing, specimens were mounted in ProLong Gold antifade reagent (Thermo Fisher Scientific) and imaged using a Leica SP8 laser scanning confocal microscope with 63 × oil-immersion objective.

### The action sites assay

To visualize the localization of USCDs in hair cells, cochlear specimens were processed for immunofluorescence analysis using myosin 7a antibody and DAPI nuclear staining, followed by examination with confocal laser scanning microscopy (CLSM). Experimental mice received an intravenous injection of 10 mg/kg USDCs and were sacrificed 6 h post-administration under anesthetization. Following dissection, cochleae were fixed, permeabilized in 1% Triton X-100 for 30 min, and blocked with 5% donkey serum for 1 h at RT. Subsequently, tissues were incubated overnight at 4 °C with anti-myosin 7a primary antibody (1:400 dilution; Cat# 25–6790, Proteus Biosciences) in blocking solution. After three washes with phosphate-buffered saline (PBS, pH 7.4), samples were incubated for 1 h at RT with species-matched Alexa Fluor-conjugated secondary antibodies (1:1000) co-administered with 10 μg/mL DAPI for nuclear counterstaining. Following three additional PBS washes, tissue sections were mounted using anti-fade mounting medium and imaged using a Leica SP8 laser scanning confocal microscope equipped with appropriate filter sets. Image acquisition parameters were maintained constant across all experimental groups to ensure comparability.

### Determination of mitochondrial ROS levels

Acute organotypic cultures of cochlear explants were established from postnatal day 5 (P5) C57BL/6 mice for live-cell imaging studies. Following euthanasia, cochleae were dissected in calcium-free D-Hanks balanced salt solution (Solarbio, Cat# H1025, Beijing, China) under sterile conditions. Through microsurgical dissection, Reissner’s membrane was carefully removed using fine forceps to expose the intact organ of Corti while preserving the basilar membrane architecture. Viable explants were maintained in DMEM/F-12 medium (Gibco, Cat# 11,330,032) containing 10% heat-inactivated fetal bovine serum (FBS; Gibco) and 150 U/mL penicillin (Sigma-Aldrich) under standard culture conditions (37 °C, 5% CO₂ humidified atmosphere) for 6 h stabilization period prior to interventions. Quality-controlled explants demonstrating intact cellular morphology (assessed via phase-contrast microscopy) and absence of mechanical damage were randomly assigned to experimental groups: (1) 1 mM H₂O₂ challenge, (2) 1 mM H₂O₂ + 10 μg/mL USCDs co-treatment, or (3) untreated controls. Following 48 h treatments, mitochondrial superoxide production was quantified using 5 μM MitoSOX^™^ Red (Invitrogen, Cat# M36008) loaded for 10 min under culture conditions. Post-staining, specimens underwent three 5-min washes with pre-warmed PBS (pH 7.4) before imaging. After washing with PBS, the specimens were visualized using a Leica SP8 laser scanning confocal microscope with 63 × oil-immersion objective.

### Intracellular iron assessment

RhoNox-6 is a novel ferrous ion red fluorescent probe and has excellent cell membrane permeability, low cytotoxicity and stability. RhoNox-6 was used to detect the intracellular iron level of HEI-OC1 cells in this study. Following various treatments, the HEI-OC1 cells were incubated with (1) 100 μM Ferrous ammonium sulfate (FAS), (2) 100 μM Erastin +1 mM 2,2’-Bipyridine, (3) 100 μM Erastin +10 μg/mL USCDs, or (4) untreated controls or 30 min at 37 °C in a 5% CO_2_ atmosphere. The confocal laser scanning microscope with 100 × oil-immersion (Leica SP8; Leica, Germany) was used to capture images.

### Lipid peroxidation assay

HEI-OC1 cells were seeded in 12-well plates and cultured overnight to ~70–80% confluence before treatment with Erastin or with the USCDs for 24 h. After treatment, cells were incubated with 2 μM C11-BODIPY 581/591 (Invitrogen) in serum-free medium at 37 °C for 30 min in the dark, washed twice with cold PBS, and harvested by gentle trypsinization. Cell pellets were resuspended in PBS containing 1% FBS, and 7-AAD was added immediately before acquisition to exclude dead cells. Flow cytometry was performed on a 488 nm laser-equipped cytometer, and the oxidized C11-BODIPY signal was collected in the FITC channel. Lipid peroxidation was quantified as mean fluorescence intensity of the FITC signal.

### The toxicity analysis of USCDs in vitro

Cell viability assessment was performed using the CCK8 assay according to standardized protocol. HEI-OC1 cells were plated in 96-well culture plates at a density of 1 × 10^5^ cells/well and maintained overnight in a humidified 37 °C incubator with 5% CO₂. After subsequent PBS washing, cells were exposed to USCDs at graded concentrations (0–160 μg/mL) in serum-free medium (100 μL/well) for 24 h at 37 °C, with seven technical replicates per concentration group. For CCK8 processing, 10 μL of CCK8 solution was added to each well together with 90 μL of medium and followed by 2 h incubation. Finally, optical density was measured at 490 nm using a microplate reader (BioTek Synergy H1), with cell viability calculated as:

$$\mathrm{Viability} \left(\%\right)= \frac{({\mathrm{OD}}_{\mathrm{sample}}-{\mathrm{OD}}_{\mathrm{blank}})}{({\mathrm{OD}}_{\mathrm{control}}-{\mathrm{OD}}_{\mathrm{blank}})}\times 100\%$$

### Hemolytic activity assay

Blood collected from C57BL/6 mice was centrifuged at 2000 g for 5 min to isolate erythrocytes, which were subsequently washed three times with PBS and resuspended. A series of dilutions of USCDs (0–160 μg/mL) was then incubated with 4% erythrocyte suspensions in 96-well plates for 1 h at 37 °C. Erythrocytes treated with PBS and 1% Triton X-100 (Solarbio, Beijing, China) served as negative and positive controls, respectively. Following centrifugation, the supernatants were collected and the absorbance at 490 nm was recorded. The hemolysis rate was calculated using the following equation:

$$\begin{aligned}&\text{Hemolysis (\%)} \\&\quad= \frac{(OD_{\mathrm{sample}} - OD_{\text{negative control}})}{(OD_{\text{positive control}} - OD_{\text{negative control}})} \\&\qquad\times 100\%\end{aligned}$$

All experiments were performed in triplicate to ensure reproducibility.

### Statistical analysis

Statistical analyses were performed using GraphPad Prism 8.0.2 (GraphPad Software, San Diego, CA). Normally distributed data are presented as mean ± standard deviation (SD). For comparisons involving three or more groups, one-way ANOVA with Tukey’s post hoc test was applied after confirming homogeneity of variances using Bartlett’s test. Pairwise comparisons between two groups were analyzed using two-tailed unpaired Student’s t-tests with Welch’s correction. All experiments consisted of three technical replicates per condition and were independently repeated with three biological replicates (n = 3). A priori significance threshold was set at *P* < 0.05, with exact p-values reported for all comparisons (ns: *p* ≥ 0.05, * p < 0.05, ** p < 0.01, *** p < 0.001, **** p < 0.0001).

## Results and discussion

### Preparation and characterizations of marine-derived CDs

**Fig. 1:**
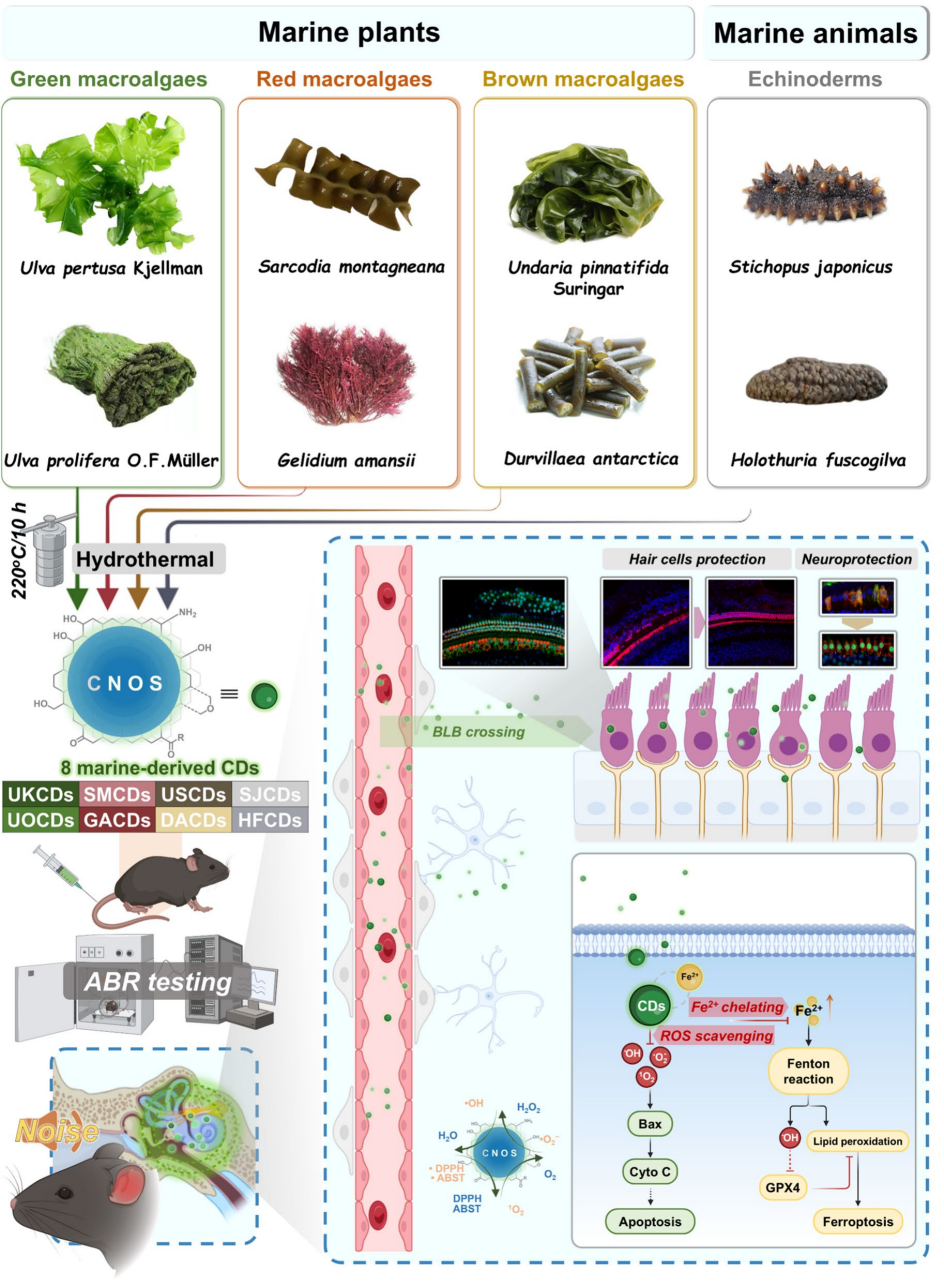
Preparation process diagram of marine-derived CDs and their mechanism of action in preventing noise-induced hearing loss. The eight marine-derived CDs were synthesized via classical hydrothermal method using macroalgae or echinoderms as carbon precursors. Among these eight CDs, the USCDs retained the greatest number of reductive functional groups (OH and NH) and showed the strongest radical scavenging activities. In the context of alleviating noise-induced hearing loss, the USCDs demonstrated the capability to inhibit hair cells apoptosis and ferroptosis through scavenging excessive ROS and ferrous ions. Moreover, the appreciable BLB crossing was fundamental for the hearing protection of USCDs in vivo

The 8 marine-derived carbon dots (UKCDs, UOCDs, SMCDs, GACDs, USCDs, DACDs, SJCDs, and HFCDs) were synthesized via the hydrothermal method using the 8 marine species (*Ulva pertusa* Kjellman, *Ulva prolifera* O.F.Müller, *Sarcodia montagneana*, *Gelidium amansii*, *Undaria pinnatifida* Suringar, *Durvillaea antarctica*, *Stichopus japonicus*, and *Holothuria fuscogilva*) as precursors, respectively (Fig. 1).

**Fig. 2:**
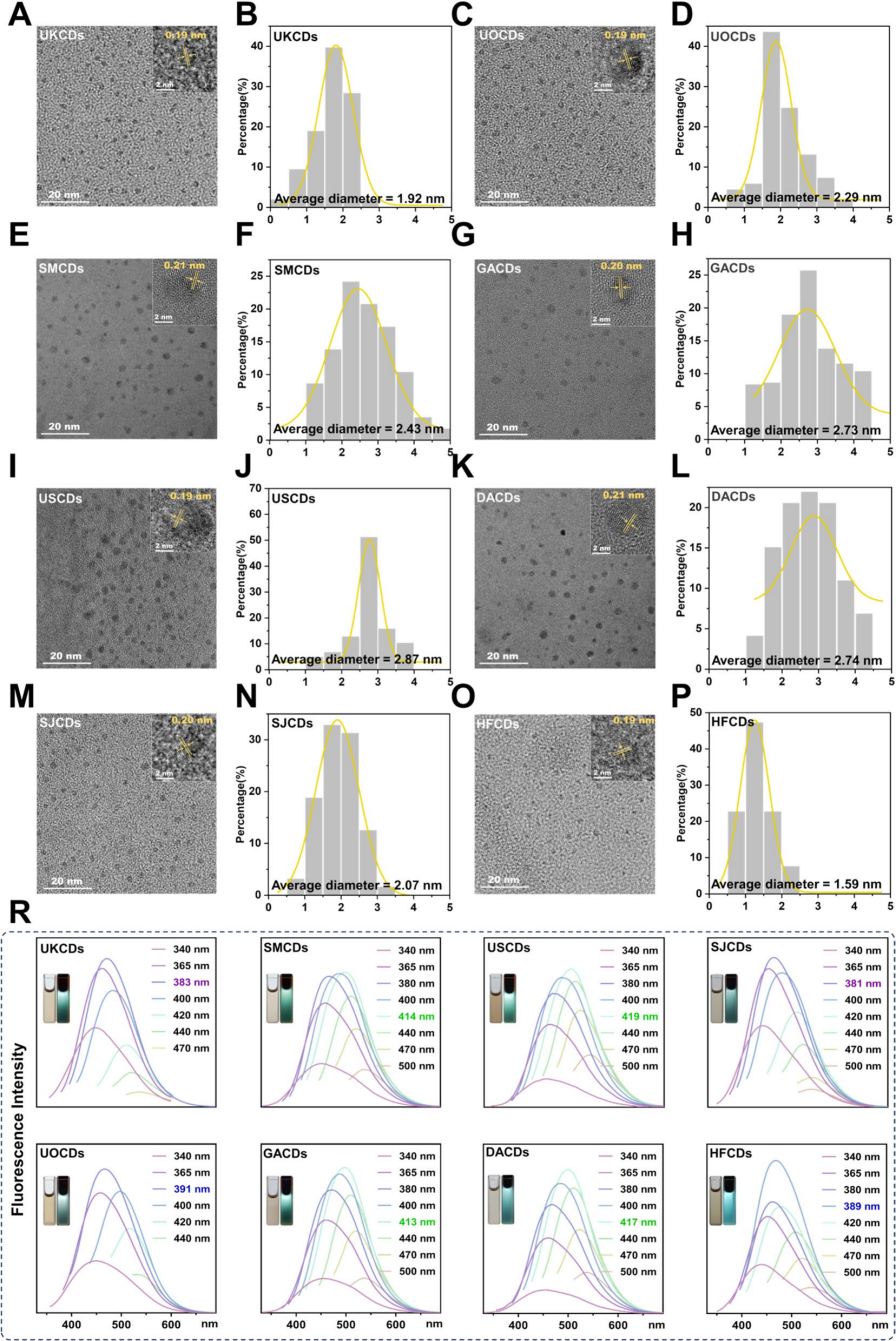
The characterization of marine-derived-CDs. (**A–P**) TEM, HR-TEM, and size distribution histogram of marine-derived CDs. (**R**) Emission spectra of marine-derived CDs under different excitations

Transmission electron microscopy (TEM) pictures of marine-derived carbon quantum dots (CDs) were obtained to confirm their shape and size distribution. Figure 2A–P illustrated that all marine-derived CDs have a spherical morphology with average diameters around 2 nm. The HRTEM images revealed crystal lattice spacings of around 0.2 nm, matching the graphite lattice plane. The zeta potentials of the marine-derived CDs were in the range of −17.5 ~ −10.3 mV (Fig. S1), indicating that the marine-derived CDs have net negative surface charges, and exert excellent colloidal dispersion and stability in aqueous solution. The photoluminescence (PL) emission spectra of the marine-derived CDs solution were analyzed to acquire comprehensive information regarding its PL characteristics. With the increase of excitation wavelengths from 340 to 500 nm, the emission peak position of the marine-derived CDs exhibits a red shift. The ideal excitation wavelengths for UKCDs, UOCDs, SMCDs, GACDs, USCDs, DACDs, SJCDs, and HFCDs are 383, 391, 414, 413, 419, 417, 381, and 389 nm, respectively (Fig. 2R). In addition, all 8 marine-derived CDs demonstrated excellent photostability in solutions under physiological pH, with negligible changes in fluorescence intensity under natural light exposure for 0–48 h, indicating good resistance to photobleaching (Fig. S2A–H).

Macroalgae and echinoderms contained abundant glycolipids, polysaccharides, and proteins with diversified functional groups (*e.g.* hydroxy, amino, amide, and carboxyl groups). Because of incomplete carbonization, some functional groups of precursors can remain in the structures of CDs. In order to analyze the chemical composition and surface functional groups of the samples, the marine-derived nano-agents were investigated by X-ray photoelectron spectroscopy (XPS) and Fourier-transform infrared (FT-IR) spectroscopy.

**Fig. 3:**
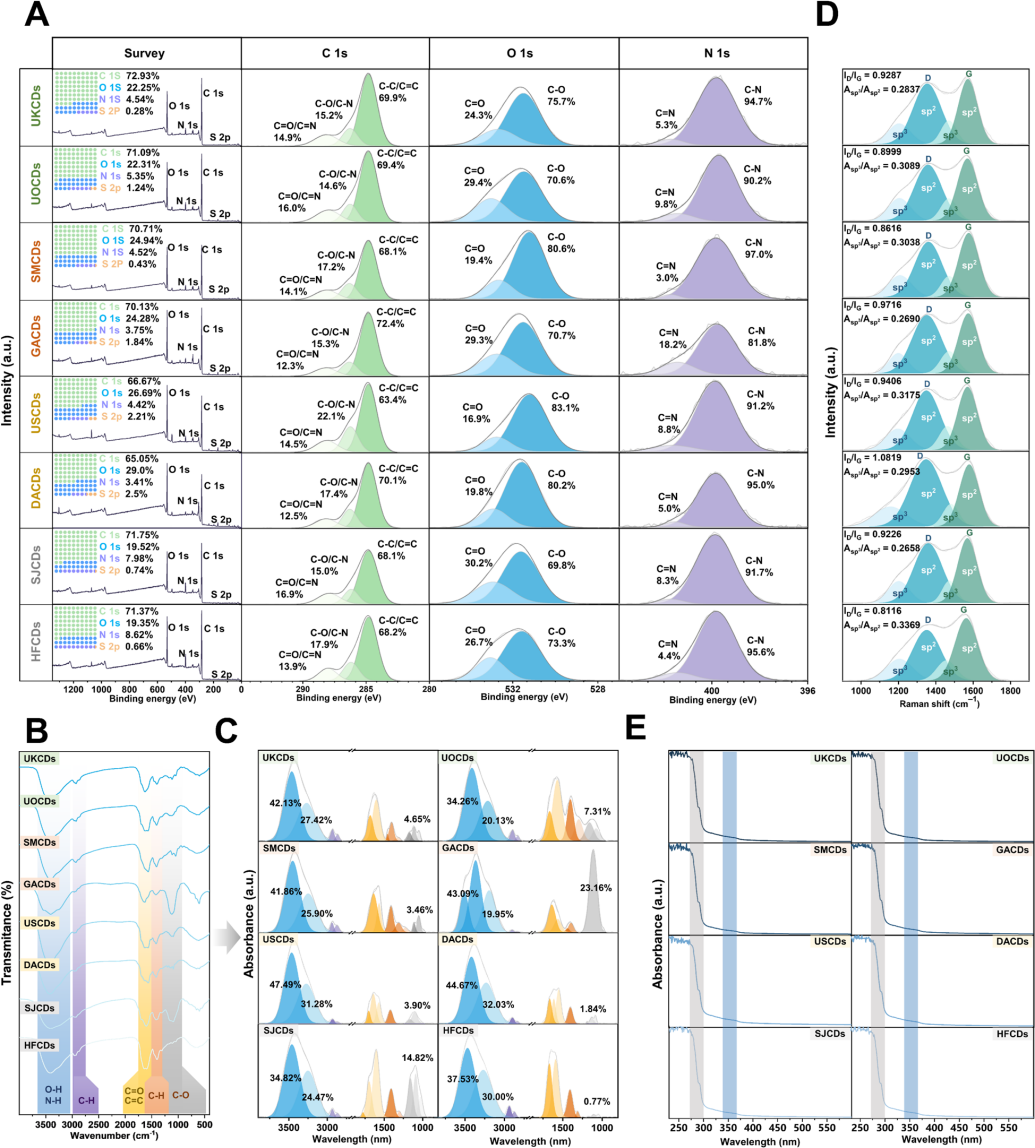
The characterization of marine-derived CDs. (**A**) XPS survey spectra, high-resolution XPS spectra of C 1 s, N 1 s, and O 1 s for marine-derived CDs. (**B**) FT-IR spectrum of marine-derived CDs. (**C**) The peaking fitting of FT-IR by Gaussian distribution. (**D**) Raman spectrum and (**E**) UV–vis spectrum of marine-derived CDs

The XPS full survey spectra revealed that C, O, N, and S atoms primarily comprise marine-derived CDs. Furthermore, the intricate bonding between C, N, and O is illustrated in the high-resolution XPS spectra. The C 1 s bands were split into three distinct Gaussian peaks centered at 284.8, 286.2, and 288.3 eV, which are assigned to graphitic C (C − C/C = C), C − O, and C = O bands, respectively. The O1s bands were deconvoluted into two Gaussian peaks centered at 531.7 and 533.0 eV for the C − O and C = O bands, respectively. The N 1 s bands consist of two Gaussian peaks centered at 399.9 and 401.8 eV for C − N and C = N bands, respectively [37, 38] (Fig. 3A).

In the FT-IR spectrum, the broad absorption peak at about 3400 cm⁻^1^ could be attributed to the stretching vibration of O–H/N–H [22, 39]. The absorption peak at about 2950 cm⁻^1^ originated from the symmetric and asymmetric stretching vibrations of C–H groups [38]. The peaks at about 1700 cm^−1^ were attributed to C = O stretching vibration [40]. The absorption peaks at about 1620 cm⁻^1^ corresponded to the absorption peak of the C = C bond in the conjugated system [41, 42]. The peaks near 1400 cm^−1^ corresponded to the bending vibration of C–H in alkyl [43]. The absorption peaks at about 1100 cm⁻^1^ were caused by the stretching vibration of the C–O bonds in hydroxyl (C–OH) or ether (C–O–C) groups [40, 44, 45] (Fig. 3B).

For in-depth analysis of structural differences among the 8 marine-derived CDs, the IR absorption peaks were deconvoluted via multi-peak Gaussian fitting [46, 47] (Fig. 3C). The relative peak intensity ratios of all absorption peaks in the wavelength region of 4000–1000 nm were calculated by integrating the areas under each peak based on IR fitting data. The strong absorption band at around 3400 cm^−1^ is attributed mainly to the stretching vibration of reductive functional groups O–H or N–H that are the crucial groups for the radical scavenging effect of CDs. As shown in Fig. 3C, the relative peak area ratios of O–H/N–H stretching (blue) of UKCDs, UOCDs, SMCDs, GACDs, USCDs, DACDs, SJCDs, and HFCDs are 69.55%, 54.39%, 67.76%, 63.04%, 78.77%, 76.70%, 59.29%, and 67.53%, respectively, which could reflect the differences in O–H/N–H content on the surface of marine CDs.

Raman spectroscopy was used to accurately measure the degree of graphitization and surface defects in marine CDs. In the Raman spectrum, the D peak around 1350 cm⁻^1^ is due to the breathing modes of sp^2^ atoms in rings and reflects the defect state of the carbon lattice, while the G peak near 1560 cm⁻^1^ is due to the bond stretching of all pairs of sp^2^ atoms in both rings and chains, indicating the graphitization level of marine CDs [48]. Here, the value of I_D_/I_G_ is a valid indicator as a measure of defect extent. Moreover, Raman bands at 1180 cm⁻^1^ and 1510 cm⁻^1^ are due to sp^3^ carbons. The vibration of sp^3^ carbon is one of the factors for the defect induced. The ratio Asp^3^/Asp^2^ can be used to measure the relative quantity of sp^3^ carbon, which reflects the degree of defects present in marine CDs. In this work, the values of I_D_/I_G_ of all samples were within the range of 0.8116 ~ 1.0819, and the values of Asp^3^/Asp^2^ were displayed in 0.2658 ~ 0.3369, which indicated that the obtained nanodots comprise partial amorphous carbon [49, 50] (Fig. 3D). The UV–vis absorption spectra of marine-derived CDs showed broad absorption in the range of 200–400 nm. This characteristic was attributed to the occurrence of π–π* and n–π* transitions within the structure of CDs. The π–π* transitions were attributed to the presence of sp^2^ hybridization in the carbon framework. The n–π* transition arises from surface functional groups like N–H, O–H, and C = O, enabling non-bonding electrons to transition to the π orbital upon photon absorption [37, 39, 51] (Fig. 3E). The XRD pattern of CDs showed broad peaks centered at about 12° and 22° (2*θ*), which correspond to amorphous carbon [38] (Fig. S3).

### Free radical scavenging evaluation of marine-derived CDs

**Fig. 4:**
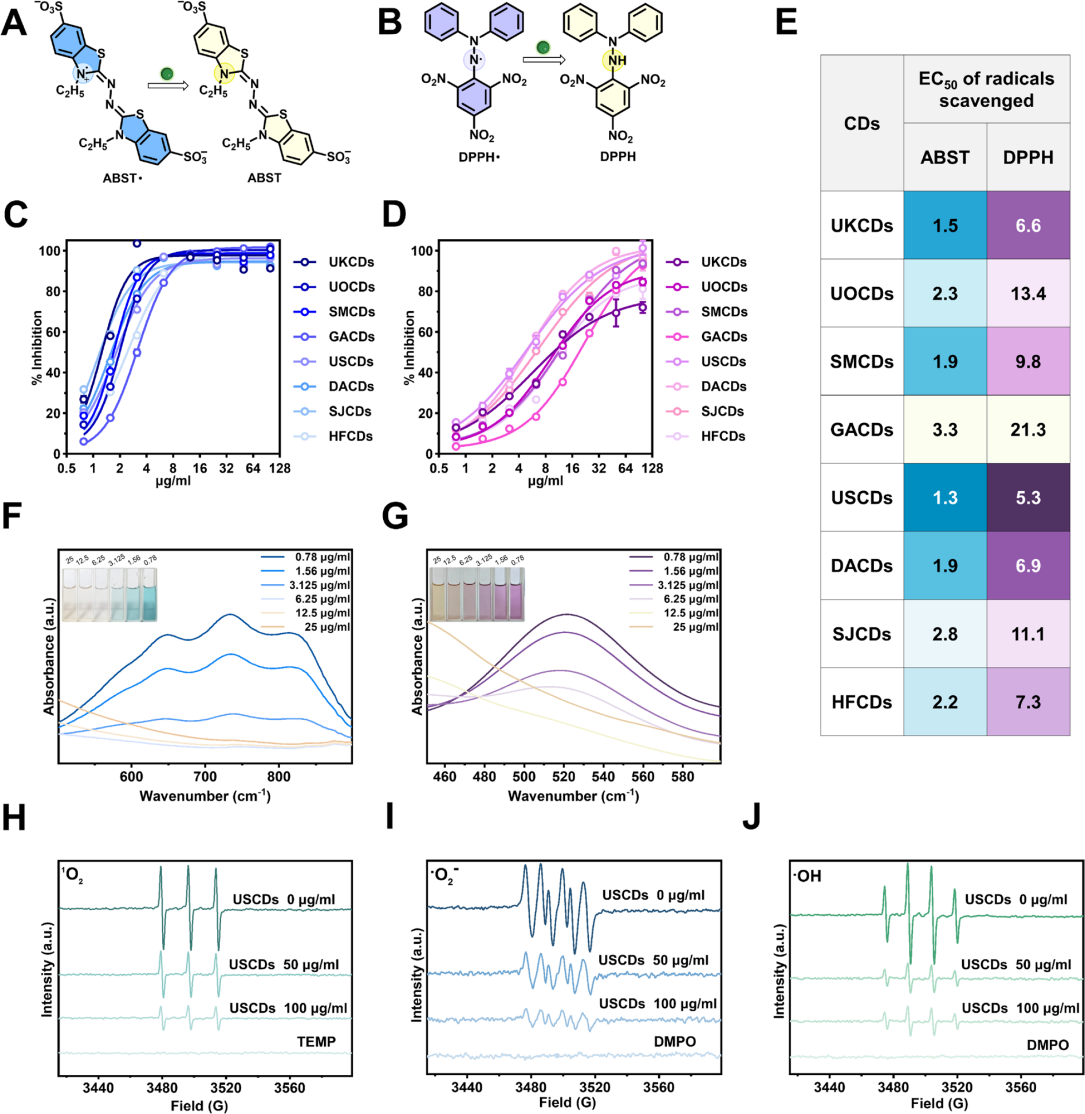
(**A**) Schematic diagram of ABST· scavenging system. (**B**) Schematic diagram of DPPH· scavenging system. (**C**) The scavenging rate of ABST· by marine-derived CDs. (**D**) The scavenging rate of ABST· and DPPH· by marine-derived CDs. (**E**) The ABST· and DPPH· scavenging EC_50_ values. UV absorption spectra of (**F**) ABST· and (**G**) DPPH· scavenged by USCDs. (**H–J**) EPR spectra detect the scavenging of ^1^O₂, ·O₂⁻, and ·OH

The excellent electron-transfer properties of CDs can endow them with outstanding catalytic activity. Many studies demonstrated that CDs derived from activated carbon possess ultrahigh free radical scavenging activities [22, 39, 51, 52]. The results of ABST and DPPH analysis indicated that the scavenging of free radicals by marine-derived CDs is concentration-dependent, with higher concentrations resulting in greater scavenging ability (Fig. 4C, D). Next, the EC_50_ values of marine-derived CDs scavenging ABST and DPPH radicals were calculated by nonlinear regression curve fit (Fig. 4E). Through surface-group capping experiments and numerous studies, it has been shown that hydroxyl (OH) and amino (NH) groups played key roles in free radical scavenging of CDs. On the basis of the IR fitting data and the EC_50_ values of radical scavenging, it was not difficult to find that the CDs exhibited stronger radical scavenging efficacy with higher relative abundance of OH/NH, and vice versa (Figs. 3C, 4E). Among these CDs, the USCDs derived from *Undaria pinnatifida* Suringar showed the strongest ABST· and DPPH· scavenging activities (EC_50_ 1.3 μg/ml against ABST·, 5.3 μg/ml against DPPH·). To probe the ROS scavenging activities of marine-derived CDs, we used electron paramagnetic resonance (EPR) technology to further detect the scavenging effect of USCDs on ^1^O₂, ·O₂⁻ and ·OH. The qualitative results indicated that USCDs have a significant scavenging effect on these three ROS, and it is concentration-dependent (Fig. 4H–J). All the above-mentioned results suggest that marine-derived CDs possess strong antioxidant capacity.

### Recovery of noise-induced auditory thresholds after marine-derived CDs treatment

**Fig. 5:**
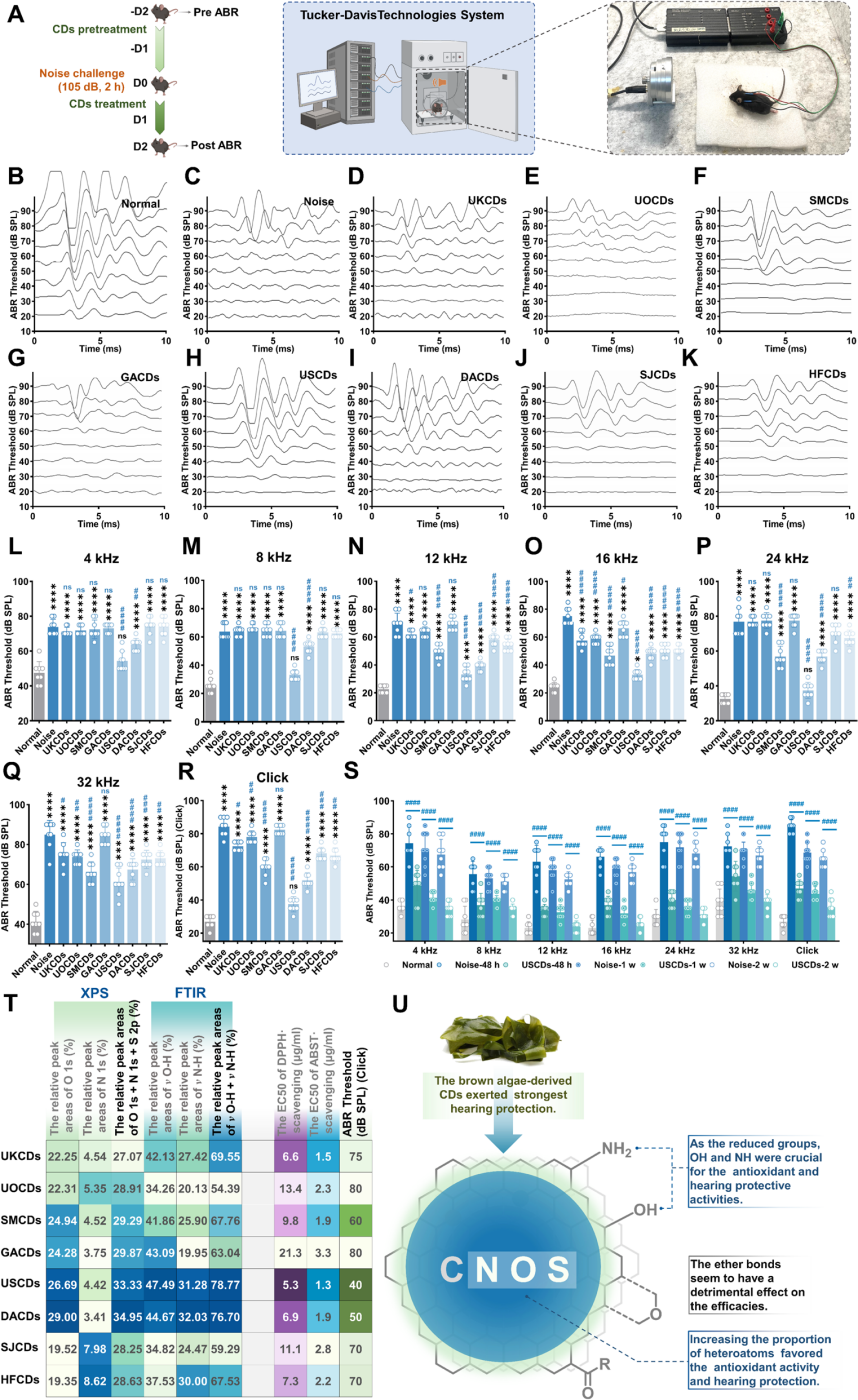
Recovery of noise-induced auditory thresholds after CDs treatment. (**A**) The overview of the noise-induced experimental design in this study. (**B**) Representative images of ABR wave recordings and thresholds in response to click sounds (*n* = 5) after no treatment (**B**), noise challenge and no treatment (**C**), noise challenge and UKCDs (**D**), UOCDs (**E**) SMCDs (**F**), GACDs (**G**), USCDs (**H**), DACDs (**I**), SJCDs (**J**), HFCDs pretreatment (**K**). Quantitative data of ABR thresholds responded to pure tone burst (*n* = 5) at 4 kHz (**L**), 8 kHz (**M**), 12 kHz (**N**), 16 kHz (**O**), 24 kHz (**P**), and 32 kHz (**Q**) frequencies for each of the treatment groups. ABR thresholds responded to click sounds (*n* = 5) after noise treatment or CDs pretreatment (**R**). (**S**) ABR thresholds were tested to respond pure burst at each frequency. CDs therapy at two weeks, one week and noise at 48 h versus controls, respectively (n = 8). CDs or noise groups *vs* normal groups * p < 0.05, **p < 0.01, *** p < 0.001, **** p < 0.0001. CDs groups *vs* noise groups ^*#*^* p* < 0.05, ^*##*^*p* < 0.01, ^*###*^* p* < 0.001, ^*####*^* p* < 0.0001. (**T**) A detailed table comparing the chemical composition of the different CDs using XPS survey and FTIR spectrums, and the radical scavenging and hearing protective effects. and (**U**) the summary of structure–activity relationship for marine-derived CDs

To determine whether CDs could protect hearing loss through intravenous administration, we constructed a model based on noise exposure. Figure 5A schematically represents the experimental timeline for pharmacological interventions in C57BL/6 mice. Both sexes of age-matched mice (8–10 weeks old) were randomly allocated into ten experimental groups receiving either CDs including UKCDs, UOCDs, SMCDs, GACDs, USCDs, DACDs, SJCDs, and HFCDs at 10 mg/kg body weight (Fig. S4), or sterile saline vehicle (0.9% NaCl) via intravenous administration. Mice underwent baseline ABR recordings (Fig. S5) followed by pretreatment with CDs for 2 d, exposure to an acoustic over-stimulation challenge (105 dB SPL for 2 h), and then continued to treat with CDs for 2 d. Figure 5B–K depicted representative ABR waveforms under different conditions.

Normal group (B) exhibited intact and well-defined waveforms, whereas noise-exposed animals (C) demonstrated significant waveform degradation and threshold elevation. Treatment with CDs derived from diverse sources (D–K; UKCDs, UOCDs, SMCDs, GACDs, USCDs, DACDs, SJCDs, HFCDs) showed differential efficacy in preserving waveform integrity, with the USCDs group closely approximating normal ABR morphology. Quantitative ABR threshold analyses across multiple test frequencies (Fig. 5L–Q) and click stimuli (Fig. 5R) further confirmed these protective effects. Noise exposure markedly elevated thresholds across all tested frequencies compared to normal group. CDs treatment, however, attenuated these threshold shifts to varying extents. In particular, USCDs demonstrated the most significant protective efficacy. As time went on, the USCDs treatment group showed further improvement in hearing loss one week after exposure to noise. After two weeks, the ABR thresholds of USCDs treatment group were closer to baseline levels at mid-to-high frequencies (Fig. 5S). These findings demonstrated that USCDs treatment alleviated hearing loss in a noise-induced mice model.

### Structure–activity relationship (SAR) analysis of marine-derived CDs

The characterization data of marine-derived CDs indicated that various chemical structures of nanoparticles were obtained from different marine precursors. We then performed a structure–activity relationship (SAR) analysis for the 8 CDs on the basis of the results of the structural characterization data and the bioactivity testing (Fig. 5T and U).

From the quantitative statistical results of survey XPS spectrums, higher heteroatom (O, N, S) content of marine-derived CDs induced their stronger ABST and DPPH radical scavenging capacities and protective efficacy from hearing loss from noise. At the same time, the reductive functional groups, including hydroxyl and amino, were critical for the bioactivities according to the results of FTIR Gaussian fitting. The USCDs and DACDs with the higher amount of heteroatom (33.33% and 34.95%, respectively) and reductive groups (relative integral area in FTIR 78.77% and 76.70%, respectively) showed the stronger biological activities in vitro (DPPH EC_50_ 5.3 and 6.9 μg/ml, ABST EC_50_ 1.3 and 1.9 μg/ml, respectively) and *in vivo* (ABR threshold/click 40 and 50 dB, respectively), while the UOCDs and GACDs with the lower amount of heteroatom (28.91% and 29.87%, respectively) and reductive groups (relative integral area in FTIR 54.39% and 63.04%, respectively) exhibited weaker radical scavenging capacities (DPPH EC_50_ 13.4 and 21.3 μg/ml, ABST EC_50_ 2.3 and 3.3 μg/ml, respectively) and hearing protection (ABR threshold/click both at 80 dB, respectively). Furthermore, the ether bonds seem to have a detrimental effect on both the radical scavenging and anti-NIHL efficacies. According to the Gaussian fitting results of FT-IR, the relative integral area near 3400 cm^−1^ (the stretching vibration of O–H/N–H) of GACDs was just 63.04%, whereas that around 1100 cm^−1^ (the stretching vibration of the C–O bonds in C–OH or C–O–C) was highest among the 8 CDs (23.16%). It suggested that GACDs have relatively higher ether bond (C–O–C) contents on the surface compared to the others. Meanwhile, the GACDs showed the lowest anti-NIHL activity *in vivo*. A similar phenomenon appeared in the SJCDs. (Figs. 3C, 5T).

In this study, the marine precursors could be classified into four major groups based on organism taxonomy information, including green algae, red algae, brown algae, and echinoderm. Among the 8 CDs, the USCDs and DACDs were the two best active anti-NIHL nanoagents, which were both derived from brown algae (*Undaria pinnatifida* Suringar and *Durvillaea antarctica*, respectively). It suggested that brown algae or their signature fractions (*e.g.* fucoidan) might be the ideal precursors for the preparation of anti-NIHL CDs. (Fig. 5U).

### USCDs exerted BLB crossing and prevented NIHL cochlear hair cells damage *in vivo*

**Fig. 6:**
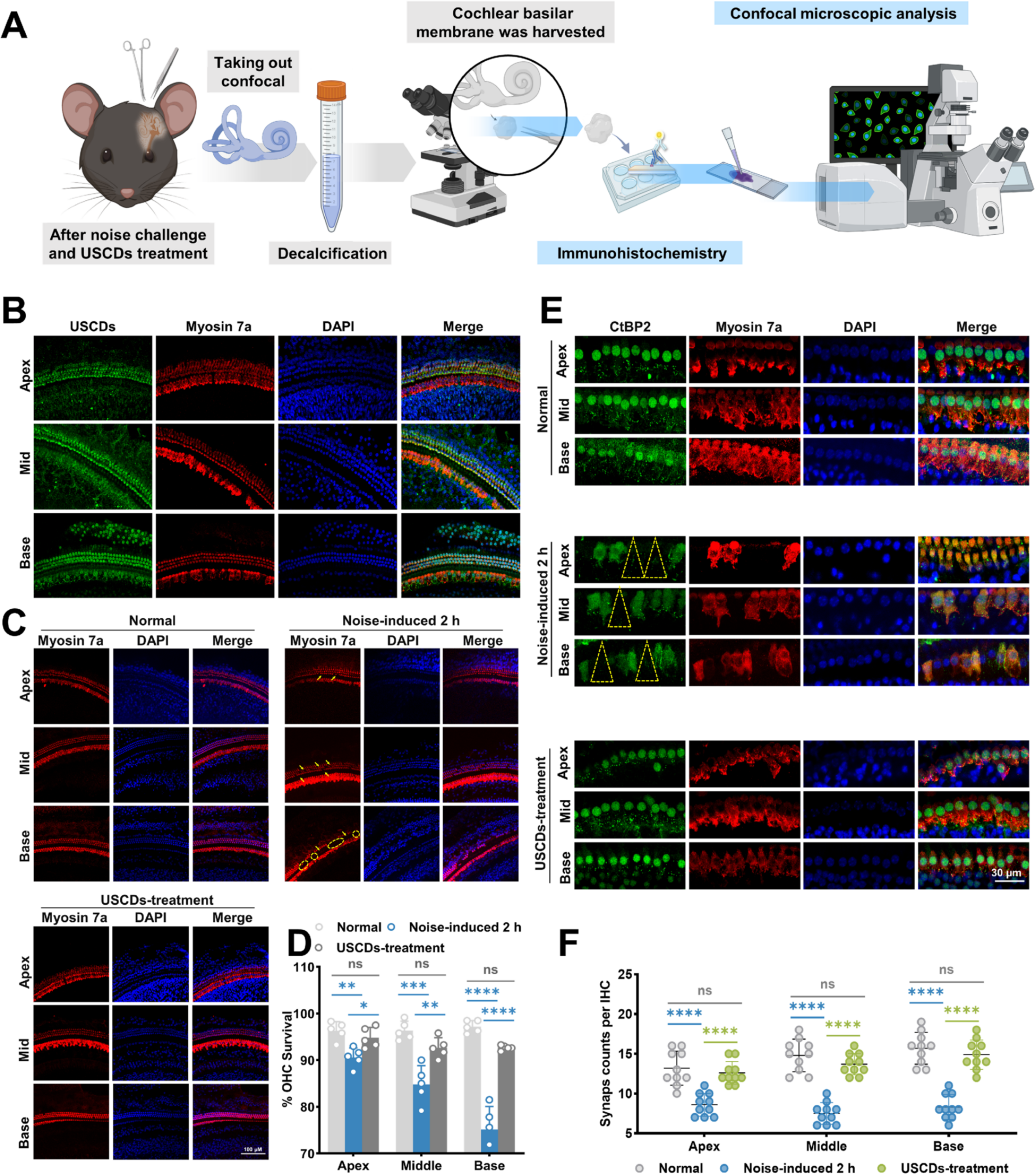
USCDs protected inner hair cell and ribbon synapses from noise-induced hearing loss. (**A**) Schematic diagram of the experimental procedure used to collect cochlear samples for confocal microscopic analysis. (**B**) Representative images of cochlear tissue. Cochlear tissue from animals treated with USCDs, showing USCDs in green, Myosin 7a in red and DAPI in blue. The merge images highlighted the colocalization of these markers, with a focus on hair cell preservation across the cochlear sections. (**C**) Comparison between normal cochlea, noise-induced damage, and USCDs pre-treatment before noise exposure. (**D**) This bar graph quantified the survival of outer hair cells (OHCs) at three different cochlear locations (apex, middle, base) across the experimental conditions: normal, noise-induced 2 h, and USCDs-pretreatment. **p < 0.01, ****p < 0.0001. (**E**) Confocal images of cochlear tissue at different locations (apex, middle, base) stained for CtBP2, Myosin 7a, and DAPI. (**F**) Bar graphs presented quantification of synapse counts per hair cell (HC) at different cochlear regions (apex, middle, base) across the experimental conditions: normal, noise-induced 2 h, and USCDs pretreatment, ****p < 0.0001

To observe whether USCDs can enter cochlear hair cells and their localization within the hair cells through intravenous injection, cochlear tissues were harvested and immediately fixed, followed by microdissection into apical, middle, and basal segments after USCDs treatment for 6 h (Fig. 6A). Hair cells (HCs) were subsequently stained with myosin7a antibody (red). Using DAPI (blue) as an internal reference, the findings indicated that the distribution pattern of USCDs (green) within HC appeared significantly enriched in the nucleus in addition to the cytoplasm (Fig. 6B). The results indicated that USCDs could move freely through the BLB into HC, which strongly suggested an important reason for the *in vivo* anti-NIHL efficacy of USCDs. Moreover, this labeling technology provided an important technical means for elucidating the structure and function of the HC at the cellular level, and exploring the cochlear pathological change.

Following ABR assessment, cochlear tissues were harvested and immediately fixed, followed by microdissection into apical, middle, and basal segments. HCs were subsequently stained with myosin7a antibody (red). Quantitative analysis revealed significant reductions in myosin7a-positive HC counts across all cochlear turns in the noise-exposed group compared with the normal group (Fig. 6C). Pretreatment with USCDs (10 mg/kg) significantly attenuated noise-induced HCs loss, particularly in the basal and middle turns, demonstrating superior protection relative to the noise-only group (Fig. 6C, D). Specifically, noise exposure induced substantial depletion of outer hair cells (OHCs) in both basal and middle cochlear regions, whereas USCDs pretreatment effectively preserved OHC integrity against acoustic trauma.

NIHL is also associated with cochlear synaptopathy, characterized by ribbon synapse degeneration. To evaluate the neuroprotective potential of USCDs, cochlear specimens were immunostained with antibodies against C-terminal binding protein 2 (CtBP2), a presynaptic ribbon marker. Confocal microscopy analysis revealed CtBP2-positive presynaptic ribbons (red signal) juxtaposed to hair cell nuclei, with synaptic puncta within inner hair cells (IHCs) demarcated by dotted-line contours (Fig. 6E). Quantitative counting demonstrated significant noise-induced reductions in CtBP2-immunoreactive puncta per IHC across all cochlear turns compared to normal group. Remarkably, USCDs treatment group attenuated noise-induced synaptic loss (Fig. 6F), with preserved synaptic density comparable to baseline levels. These findings established USCDs as effective protectors against noise-induced synaptopathy through by maintaining the integrity of cochlear ribbon synapses.

### The mechanism of USCDs preventing NIHL cochlear hair cells damage

**Fig. 7:**
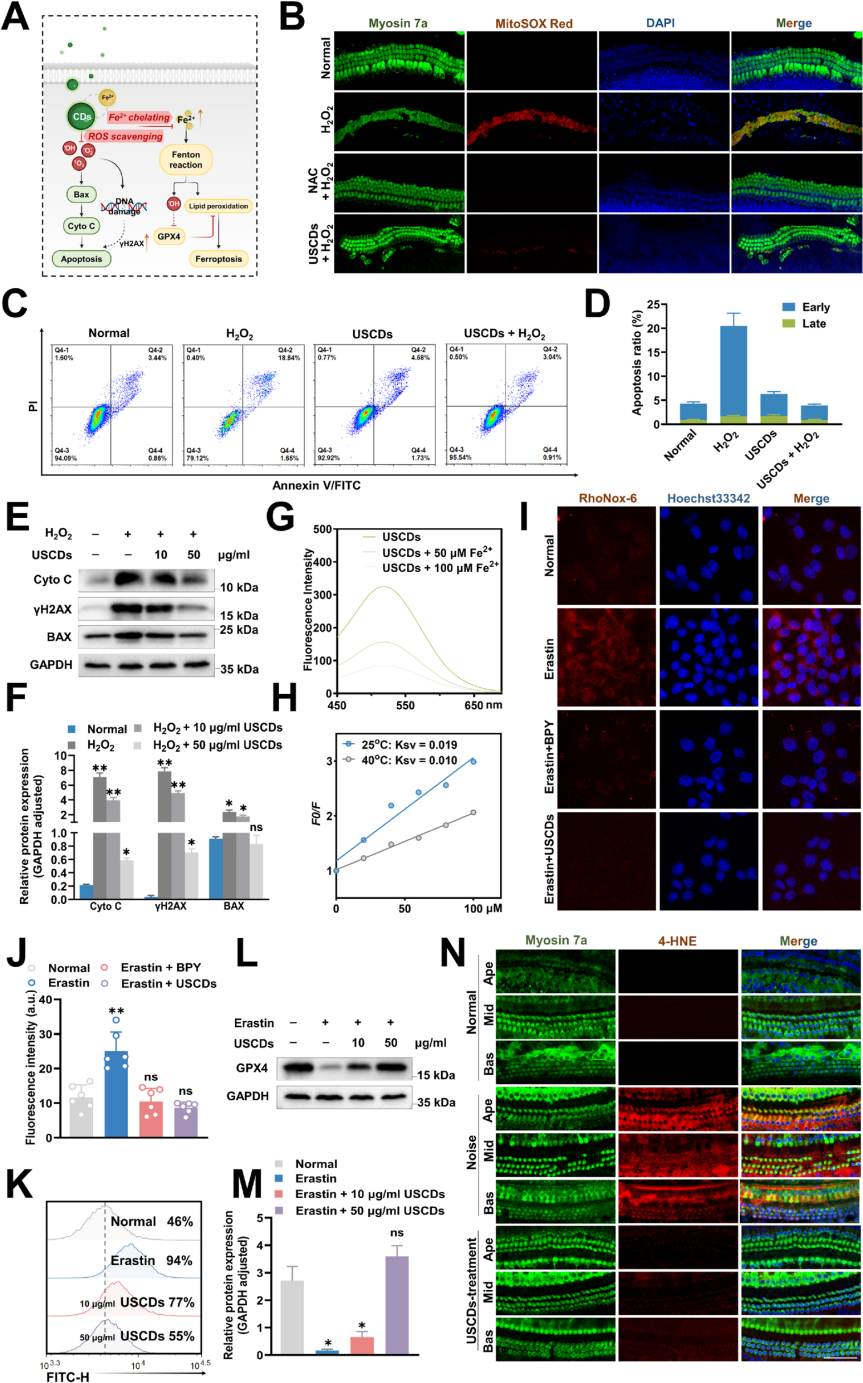
(**A**) Overview of the cytoprotective mechanism of action of the USCDs (**B**) Representative images of cochlear tissue stained for Myosin 7a, MitoSOX^™^ Red and DAPI. (**C-D**) Flow cytometry results of apoptosis under different conditions. (**E**) Western blotting and (**F**) semi-quantitative analysis of cyto c, γH2AX and Bax in HEI-OC1 cells with different treatments at 48 h, GAPDH were used as internal references. (**G**) Fluorescence emission spectra of USCDs with different concentrations of Fe^2+^ under 25 °C. (**H**) Stern–Volmer values (Ksv) of Fe^2+^ at the emission wavelengths of 419 nm. (**I**) Representative images (RhoNox-6 staining) of intracellular erastin-induced Fe^2^⁺ production in HEI-OC1 cells after different treatments. (**J**) The bar graph quantified the fluorescence intensity of RhoNox-6 in the experimental condition. (**K**) Flow cytometry analysis of intracellular lipid peroxidation in HEI-OC1 cells with different treatments. (**L**) Western blotting and (**M**) semi-quantitative analysis of GPX4 in HEI-OC1 cells with different treatments at 48 h, GAPDH were used as internal references. (**N**) The increase in 4-HNE fluorescence intensity in noise-treated hair cells of cochlear was markedly inhibited by pretreatment with USCDs in mice (n = 5). Scale bar = 50 μm. CDs groups *vs* model group * p < 0.05, ** p < 0.01, *** p < 0.001, **** p < 0.0001

In the pathogenesis of noise-induced HCs injury, excessive ROS accumulation plays a pivotal role in triggering cochlear HCs apoptosis (Fig. 7A). Oxidative stress is a definite factor for NIHL. To investigate the possible role of USCDs in resisting oxidative stress, we employed MitoSOX Red staining for mitochondrial superoxide anion radicals (·O₂⁻) detection in cochlear explants (Fig. 7B). HCs populations exhibiting co-localization of MitoSOX Red and myosin7a immunoreactivity were identified as ROS-overloaded cells, which were subsequently quantified through systematic stereological analysis.

Quantitative evaluation demonstrated that USCDs pretreatment (10 μg/mL) significantly attenuated the prevalence of ROS-positive HCs compared to the H_2_O_2_-exposed group, while normal specimens showed minimal co-labeling signals (Fig. 7B, S6). These findings collectively suggested that USCDs exerted protective effects through suppression of ROS overproduction in cochlear sensory epithelia. Next, we investigated the role of USCDs on apoptosis through Annexin V/PI in a model of H_2_O_2_-induced oxidative stress in cells. As shown in Fig. 7C and D, there was no significant difference in the apoptosis rate of cells between the normal group and the USCDs pretreatment group alone. The total apoptosis rate was increased to 20.49% after treatment with 1 mM H_2_O_2_, whereas it was reduced to 6.31% after pretreatment with USCDs. Western blotting results demonstrated that USCDs inhibited apoptosis through downregulation of proapoptotic activator Bax, and then reduced the cytochrome c (cyto c) release. Furthermore, the downregulation of the DNA damage marker γH_2_AX suggested that USCDs can protect hair cells from ROS-induced the DNA damage. (Fig. 7E, F).

The abundant hydroxyl and amino groups on the surface of USCDs inspire us to explore their binding properties with Fe^2+^. The intrinsic fluorescence intensity (FI) of USCDs decreases continuously by increasing the concentration of the metal ion (Fe^2+^) and the Stern–Volmer value decreases as growing the temperature, indicating an interaction between them (Fig. 7G, H). Intracellular ferrous ion (Fe^2^⁺) levels in HEI-OC1 cells were assessed using the fluorescent probe RhoNox-6. Under physiological conditions, control cells exhibited weak red fluorescence, indicating the presence of basal levels of endogenous Fe^2^⁺. Treatment with Erastin for 30 min resulted in a significant increase in intracellular Fe^2^⁺, as evidenced by a marked enhancement of RhoNox-6-derived red fluorescence. Subsequent administration of BPY, a ferrous ion chelator, effectively sequestered Fe^2^⁺ and prevented its reaction with RhoNox-6, leading to a pronounced reduction in the fluorescent signal. The application of USCDs similarly reduced the fluorescence intensity to an extent comparable to the BPY treatment group. This observation suggested that USCDs effectively decreased the Fe^2^⁺ accumulation level induced by Erastin (Fig. 7I, J). The USCDs exhibited a concentration-dependent protective effect against Erastin-induced ferroptosis in HEI-OC1 cells, as evidenced by a progressive reduction in C11-BODIPY fluorescence intensity with increasing drug concentrations (Fig. 7K). Decreased GPX4 is a hallmark of ferroptotic cell death. Western blotting results indicated that USCDs reversed Erastin-induced downregulation of GPX4, whose inhibitory effect appeared to be dose dependent (Fig. 7L, M). To further detect the effect of USCDs on Erastin-induced ferroptosis, a CCK8 assay was conducted. The results clearly demonstrated that USCDs reversed the Erastin-induced decrease in cell viability (Fig. S7).

Immunofluorescence staining was performed to evaluate oxidative stress and Ferroptosis inhibition in cochlear hair cells following noise exposure by measuring 4-hydroxynonenal (4-HNE) in vivo, a well-established marker of lipid peroxidation. Under normal conditions, no detectable 4-HNE signal was observed in cochlear hair cells. Following noise exposure led to a rise in 4-HNE levels, indicating noise-induced oxidative damage in a severity-dependent manner. Notably, treatment with USCDs significantly attenuated the noise-induced accumulation of 4-HNE. In the USCD-treatment group, the 4-HNE signal was markedly reduced compared to the noise-exposed group without treatment, suggesting a protective effect of USCDs against lipid peroxidation in hair cells following noise exposure (Fig. 7N). The observed accumulation of 4-HNE provides direct evidence that noise overexposure induces severe oxidative damage within cochlear hair cells, specifically through the peroxidation of membrane lipids. This finding is consistent with the established model of NIHL, where metabolic stress leads to an overproduction of ROS and a consequent breakdown of cellular redox homeostasis.

As a terminal and stable product of lipid peroxidation, 4-HNE generation is not merely a passive marker but is mechanistically driven by redox-active iron. The canonical pathway involves the catalysis of hydroxyl radical formation via the Fenton reaction (Fe^2^⁺ + H₂O₂ → Fe^3^⁺ + •OH + OH⁻), where labile ferrous iron (Fe^2^⁺) acts as the primary catalyst to convert ROS into highly destructive radicals that attack polyunsaturated fatty acids. Therefore, the robust suppression of 4-HNE by USCDs strongly implies an intervention upstream of lipid peroxidation, potentially through the modulation of intracellular labile iron pools. These above results obviously illustrated that the USCDs inhibited the apoptotic pathway through ROS scavenging and reduced the ferroptosis by ferrous ion chelating, which prevented the NIHL cochlear hair cell damage.

### The biosafety analysis of USCDs

**Fig. 8:**
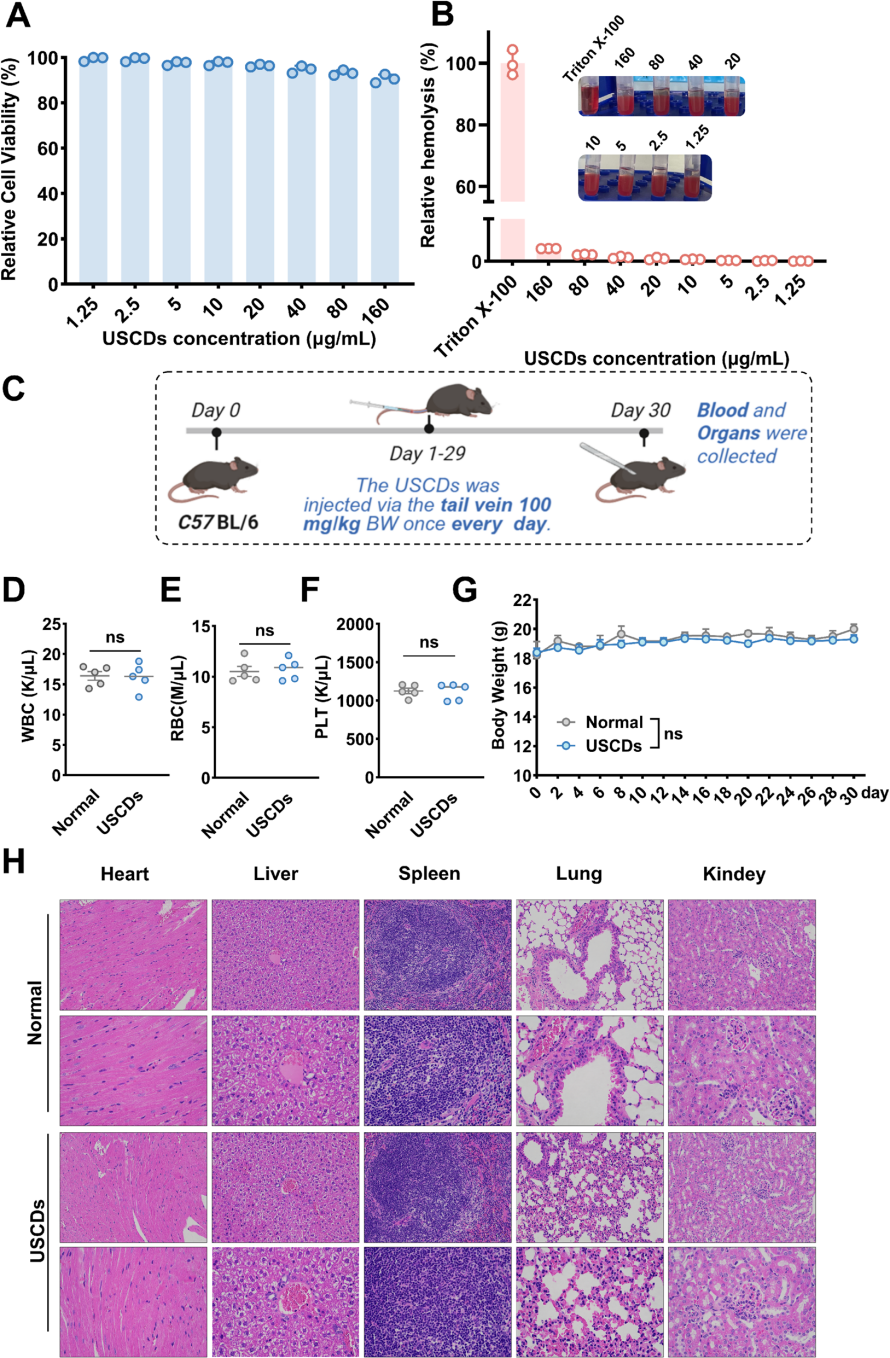
(**A**) The cell viability assay results to assess the cytotoxicity of USCDs on HEI-OC1 cells at various concentrations. (**B**) Erythrocyte hemolysis percentage of the USCDs at varying concentration. The photograph on the right showed the appearance of the hemolysis assay at different concentrations, with increasing concentrations of USCDs (from 160 μg/mL to 1.25 μg/m). (**C**) Biosafety verification scheme of USCDs in vivo. (**D–F**) The hematological parameters in the blood of mice with different treatments. (**G**) The weight of the mice was recorded in 30 days for different treatments (*n* = 5). (**H**) Histopathological images from H&E staining of major internal organs (heart, liver, spleen, lung, kidney) from animals treated with PBS or USCDs

Prior to therapeutic application evaluation, cytocompatibility assessment of USCDs was conducted using the CCK8 assay in HEI-OC1 auditory cells. Following 24 h exposure to escalating USCDs concentrations (0–160 μg/mL), dose–response analysis revealed a progressive reduction in cell viability, maintaining > 80% survival even at the maximum tested concentration (160 μg/mL) (Fig. 8A). Notably, no statistically significant cytotoxicity was observed below 160 μg/mL (*p* > 0.05 vs untreated controls). This favorable biosafety profile confirmed USCDs’ suitability for inner ear therapeutics and further substantiates their potential as innovative candidates for noise-induced hearing loss intervention. To further evaluate the therapeutic potential of USCDs, an erythrocyte hemolysis assay was performed. Erythrocytes were incubated with varying concentrations of USCDs (0–160 μg/mL) for 30 min. Dose–response analysis showed a gradual increase in hemolysis with increasing concentrations. Remarkably, even at 160 μg/mL, the hemolysis rate remained below 7% (Fig. 8B), while the hemolysis rate in the Triton X-100 group was almost 100%. These results indicated that USCDs possessed favorable biosafety *in vitro*.

To evaluate the systemic toxicity of USCDs administration, we performed comprehensive histopathological examinations on major murine organs, including the heart, liver, spleen, lungs, and kidneys, following tail vein injection (100 mg/kg BW, once daily for 30 days). Blood analysis indicated that no difference in blood cells was noted between USCDs and normal groups, confirming that the USCDs treatment had no effect on blood cell counts (leukocytes WBC, erythrocytes RBC, and platelet PLT count) (Fig. 8D–F).

The analysis of body weight curves indicated that USCDs had no effect on body weights (Fig. 8G). Next, tissue samples were collected, fixed in 4% paraformaldehyde, and subjected to histopathological analysis using hematoxylin and eosin staining. Microscopic evaluation revealed preserved tissue architecture with no evidence of inflammatory infiltrates, hemorrhagic lesions, or necrotic changes in any examined organs (Fig. 8H). These findings collectively demonstrated that intravenous administration of USCDs did not induce detectable subacute toxicity or histopathological alterations in vital organs, indicating favorable biocompatibility and potential suitability for biomedical applications.

## Conclusion

This study presented the innovative design of biocompatible and highly safe marine-derived carbon dots (CDs), derived from marine macroalgae or echinoids, as effective agents for the protection of inner ear hearing loss, particularly in noise-induced hearing loss (NIHL). Our findings demonstrated that marine-derived CDs, especially USCDs, exhibited significant auditory protection through their radical-scavenging properties and ability to cross the blood-labyrinth barrier (BLB), which is crucial for therapeutic efficacy in inner ear diseases. Structure–activity relationships suggested that improving the content of heteroatoms and hydroxyl/amino groups in the CDs played the crucial roles in radical-scavenging and anti-NIHL activities. Among the 8 marine-derived nanodrugs, the USCDs contained the largest number of heteroatoms and reduced groups (OH/NH), and showed the strongest efficiencies in radical scavenging and hearing protection. Mechanism studies suggested that USCDs decreased the critical regulators of apoptosis, including cyto c, γH2AX, and Bax, through ROS scavenging. At the same time, USCDs could suppress lipid peroxidation by reversing Erastin-induced intracellular Fe^2+^ accumulation and GPX4 degradation and then inhibit the ferroptosis of hair cells. It was important to mention that the USCDs could cross the BLB into hair cells upon systemic administration, which was identified as an urgent clinical need in treating these inner ear diseases, a major limitation for conventional therapeutics. USCDs represented a promising candidate for NIHL treatment, offering insights into the development of new therapeutic strategies derived from marine resources. This research broadens the use of biomass-derived CDs and provides opportunities for their application in various biomedical fields. With their remarkable safety profile and strong therapeutic effects, USCDs could play a significant role in mitigating the impact of noise-induced auditory damage and advancing the field of hearing loss therapeutics. To the best of our knowledge, this is the first case of anti-NIHL CDs, which is of great significance in the exploitation of marine resources, broadens the application scenarios of bioactive CDs, and is the development of a new approach for NIHL treatment.

## Supplementary Information

Additional file 1.

## Data Availability

All data supporting the findings of this study are available within the paper and its Supplementary Information.
