# Supplementary material for: Marine-derived carbon dots: a safe and effective solution for noise-induced hearing loss
Source: J Nanobiotechnology. 2026 Mar 16;24:387. doi: 10.1186/s12951-026-04282-9 (PMC13104310; doi:10.1186/s12951-026-04282-9)
Supplement: Supplementary file 1 — Additional file 1. [file 12951_2026_4282_MOESM1_ESM.doc]

**Supplemental files for**

**Marine-****Derived Carbon Dots: A Safe and Effective Solution for Noise-Induced Hearing Loss**

Guangsen Xu^1*^, Jing Wang^1^, Guige Hou^1^, Xiaoya Wang^1^, Yuliang Xu^2^, Jiajun Tian^3*^, Yanjiao Ding^3, 4*^

*^1^ School of Pharmacy, the Key Laboratory of Prescription Effect and Clinical Evaluation of State Administration of Traditional Chinese Medicine of China, Binzhou Medical University, Yantai, 264003, PR China*

*^2^Department of Pharmacy, The Second Qilu Hospital of Shandong University, 250033, 247 Beiyuan Street, Jinan, Shandong, PR China*

*^3^ Department of Otolaryngology-Head and Neck Surgery, Shandong Provincial ENT Hospital, Shandong University; Jinan, Shandong, PR China.*

*^4^ Department of Pharmacy, Shandong Second Provincial General Hospital; Jinan, Shandong, PR China.*

*Corresponding authors

1. Yanjiao Ding

Department of Pharmacy, Shandong Second Provincial General Hospital; Jinan, Shandong, China.

E-mail: dingyanjiao@email.sdu.edu.cn

2. Jiajun Tian

Department of Otolaryngology-Head and Neck Surgery, Shandong Provincial ENT Hospital, Shandong University; Jinan, Shandong, China.

E-mail: [tianjiajun212@163.com](mailto:tianjiajun212@163.com)

3. Guangsen Xu

School of Pharmacy, the Key Laboratory of Prescription Effect and Clinical Evaluation of State Administration of Traditional Chinese Medicine of China, Binzhou Medical University, Yantai, 264003, PR China

E-mail: [shi.heng2009@163.com](mailto:shi.heng2009@163.com)


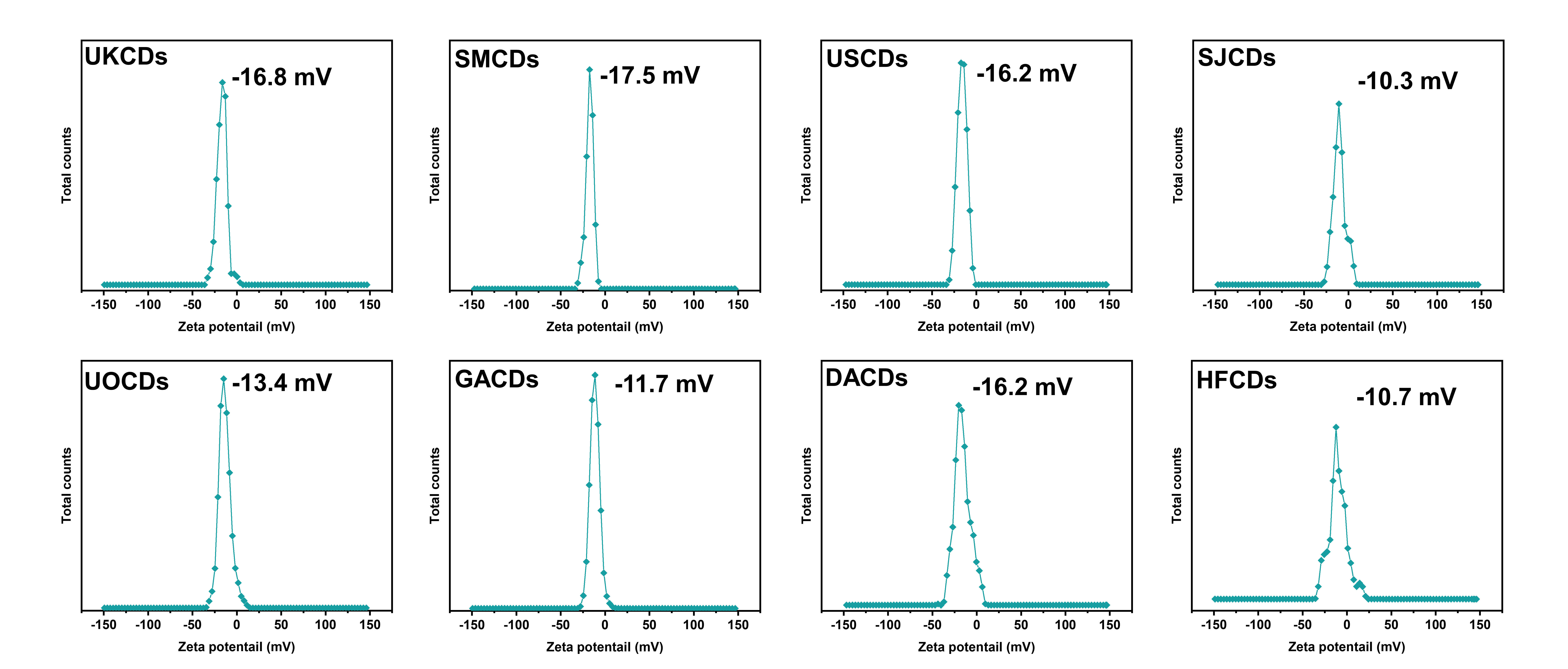


**Fig. S1.** Zeta potential of marine-derived carbon dots.


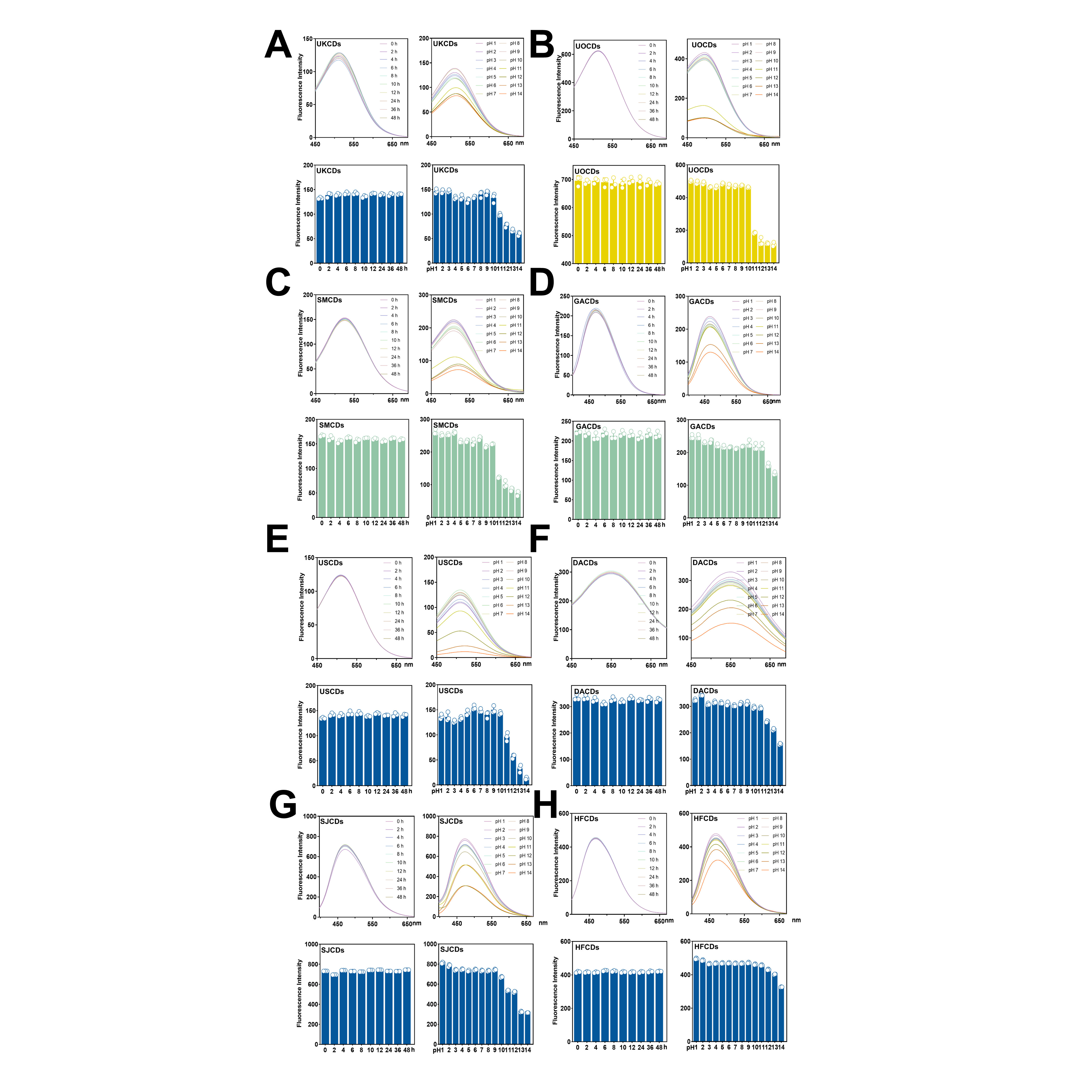


**Fig. S2.** Fluorescence emission spectra and fluorescence intensity of marine-derived CDs in the presence of aqueous solution with different pH, and different times under the light.


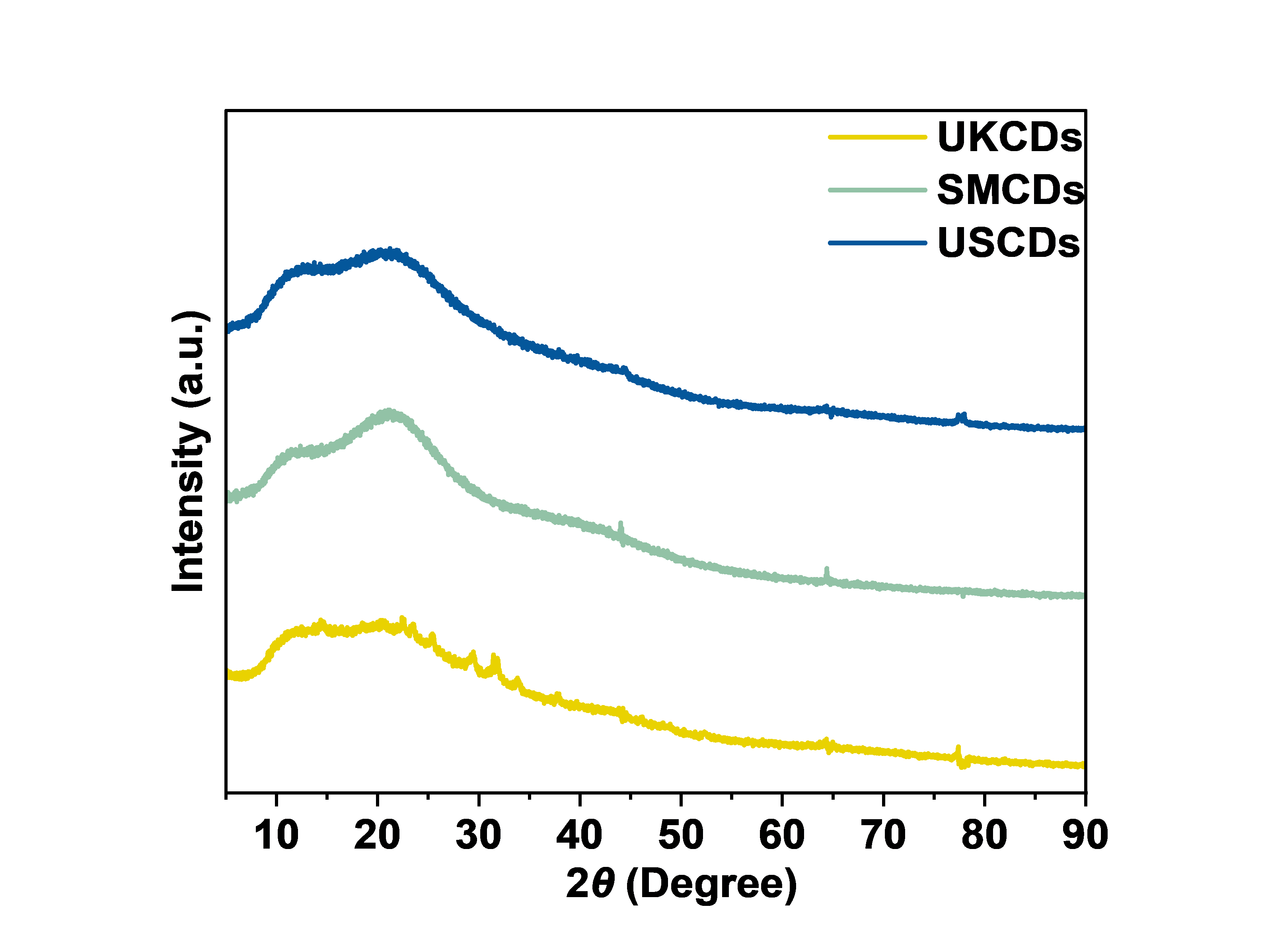


**Fig. S3.** XRD pattern of the marine-derived CDs





**Fig. S4** The baseline auditory brainstem response (ABR) thresholds (in dB SPL) were presented at different frequencies (from 4 kHz to 32 kHz) for mice after intravenous injection of different concentrations of USCDs. Each point showed the threshold response at a specific frequency for the corresponding condition.


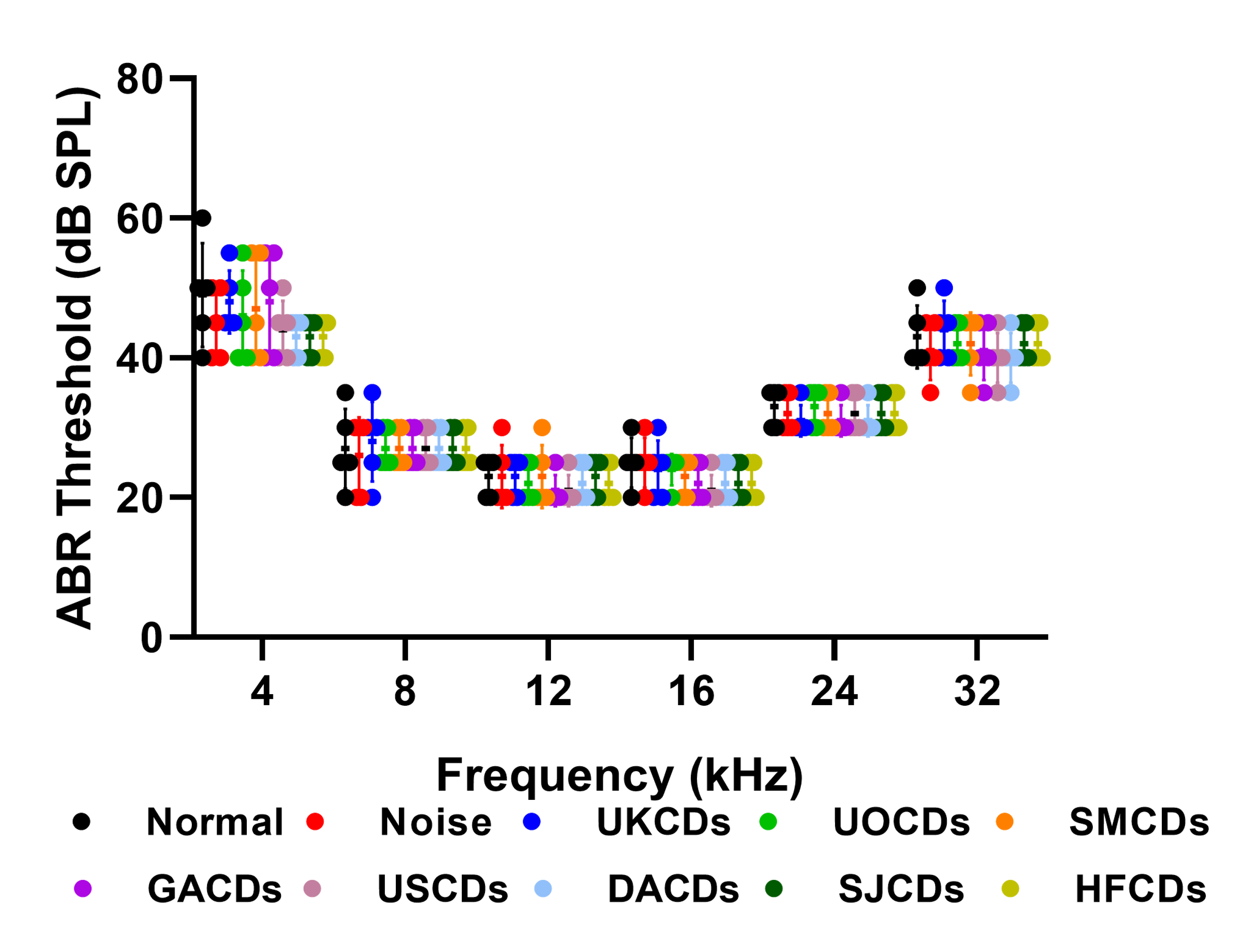


**Fig. S5** The baseline auditory brainstem response (ABR) thresholds (in dB SPL) were presented at different frequencies (from 4 kHz to 32 kHz) for mice after intravenous injection of eight different carbon dot (CD) types. Each point showed the threshold response at a specific frequency for the corresponding condition.

**Fig. S6.** The bar graph quantified the fluorescence intensity of MitoSOX™ Red (red, a mitochondrial ·O₂⁻ ROS indicator) in the experimental conditions. The y-axis represented fluorescence intensity (arbitrary units, a.u.). Statistical significance is indicated by *****p* < 0.0001.

**Fig. S7.** Effect of USCDs on the Erastin-induced ferroptosis of HEI-OC1 observed in CCK8 assay. Statistical significance is indicated by ***p* < 0.01, ****p* < 0.001.


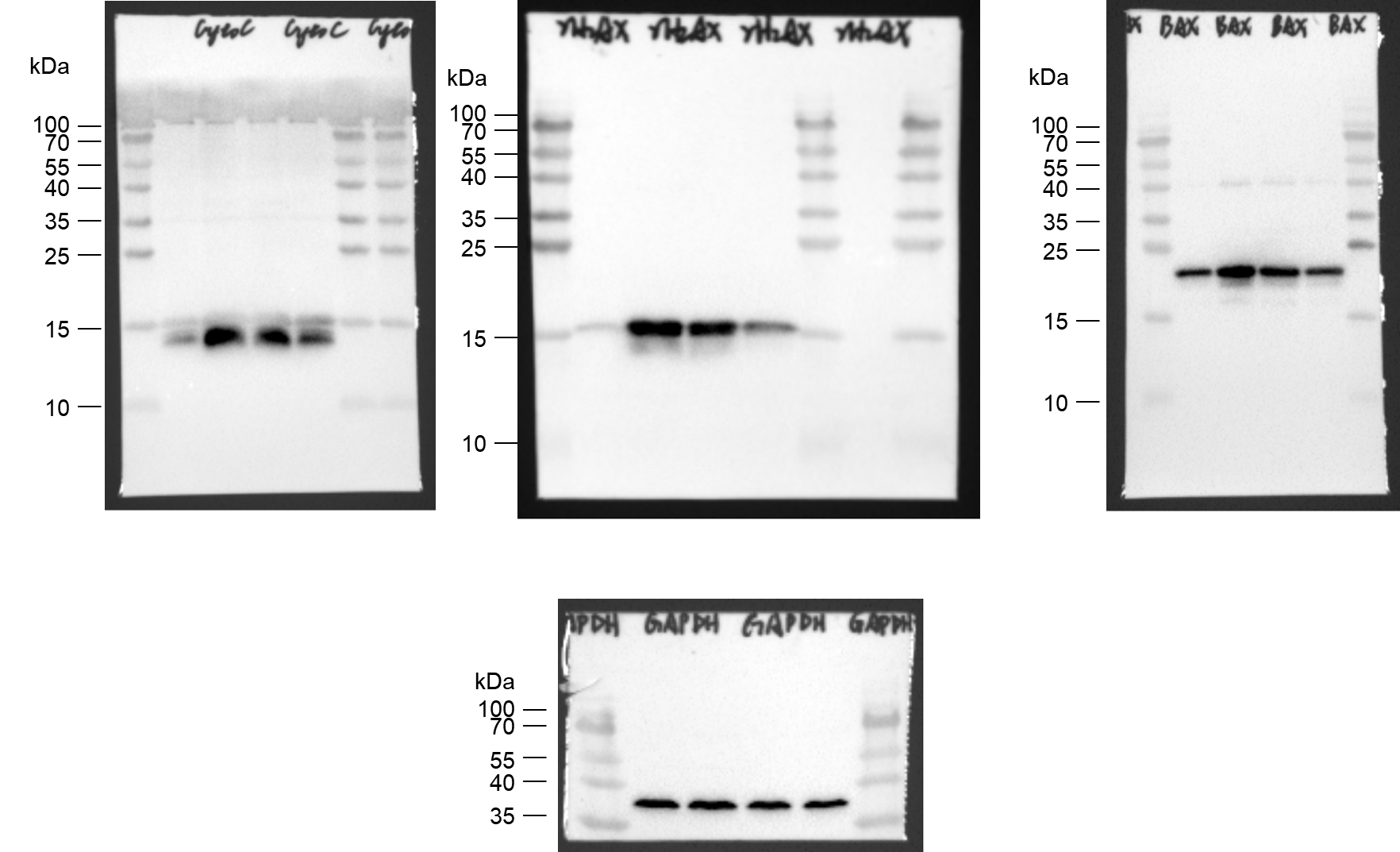


**Fig. S8.** The full uncropped Gels and Blots image(s) of **Fig. 7E**.


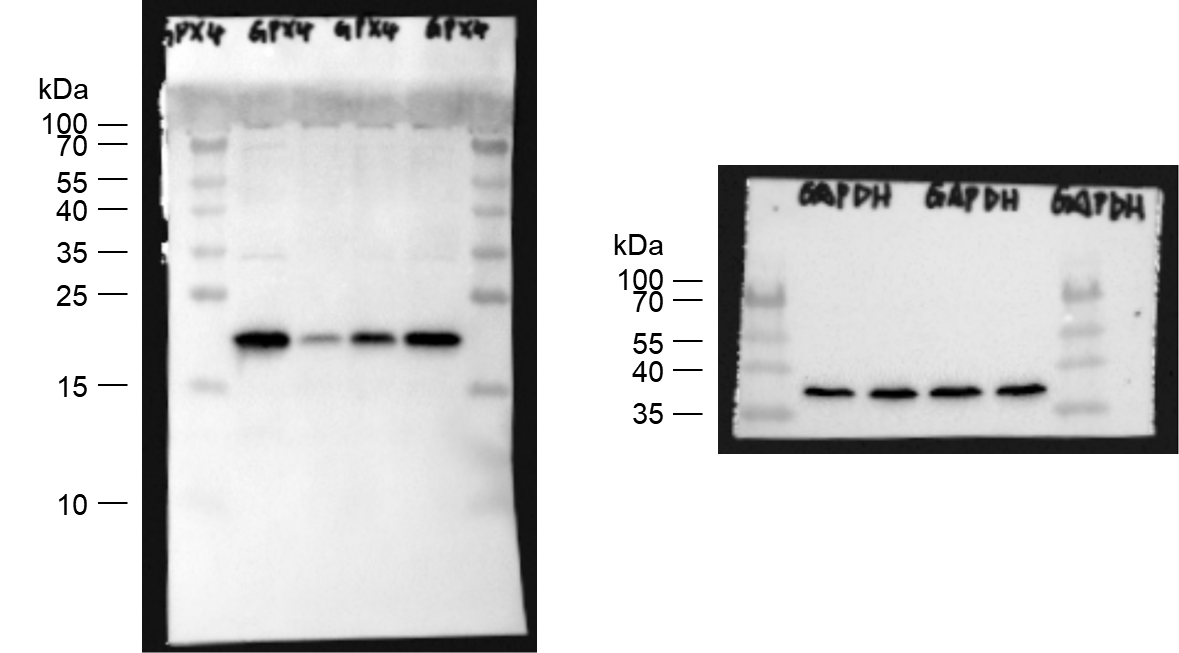


**Fig. S9.** The full uncropped Gels and Blots image(s) of **Fig. 7L**
